# Reversible Oxidation of a Conserved Methionine in the Nuclear Export Sequence Determines Subcellular Distribution and Activity of the Fungal Nitrate Regulator NirA

**DOI:** 10.1371/journal.pgen.1005297

**Published:** 2015-07-01

**Authors:** Andreas Gallmetzer, Lucia Silvestrini, Thorsten Schinko, Bernd Gesslbauer, Peter Hortschansky, Christoph Dattenböck, María Isabel Muro-Pastor, Andreas Kungl, Axel A. Brakhage, Claudio Scazzocchio, Joseph Strauss

**Affiliations:** 1 Fungal Genetics and Genomics Unit, Department of Applied Genetics and Cell Biology, BOKU—University of Natural Resources and Life Science, Vienna, Vienna, Austria; 2 Institute of Pharmaceutical Sciences, Karl-Franzens-University Graz, Graz, Austria; 3 Department of Molecular and Applied Microbiology, Leibniz Institute for Natural Product Research and Infection Biology, Hans Knoll Institute, Jena, Germany; 4 Department of Microbiology and Molecular Biology, Friedrich Schiller University Jena, Jena, Germany; 5 Health and Environment Department, Austrian Institute of Technology GmbH—AIT, University and Research Center Tulln, Tulln an der Donau, Austria; 6 Instituto de Bioquímica Vegetal y Fotosíntesis, Seville, Spain; 7 Department of Microbiology, Imperial College, London, United Kingdom, and Institute for Integrative Biology of the Cell (I2BC), CEA, CNRS, Université Paris-Sud, Orsay, France; University of Melbourne, AUSTRALIA

## Abstract

The assimilation of nitrate, a most important soil nitrogen source, is tightly regulated in microorganisms and plants. In *Aspergillus nidulans*, during the transcriptional activation process of nitrate assimilatory genes, the interaction between the pathway-specific transcription factor NirA and the exportin KapK/CRM1 is disrupted, and this leads to rapid nuclear accumulation and transcriptional activity of NirA. In this work by mass spectrometry, we found that in the absence of nitrate, when NirA is inactive and predominantly cytosolic, methionine 169 in the nuclear export sequence (NES) is oxidized to methionine sulfoxide (Met^ox^169). This oxidation depends on FmoB, a flavin-containing monooxygenase which *in vitro* uses methionine and cysteine, but not glutathione, as oxidation substrates. The function of FmoB cannot be replaced by alternative Fmo proteins present in *A*. *nidulans*. Exposure of *A*. *nidulans* cells to nitrate led to rapid reduction of NirA-Met^ox^169 to Met169; this reduction being independent from thioredoxin and classical methionine sulfoxide reductases. Replacement of Met169 by isoleucine, a sterically similar but not oxidizable residue, led to partial loss of NirA activity and insensitivity to FmoB-mediated nuclear export. In contrast, replacement of Met169 by alanine transformed the protein into a permanently nuclear and active transcription factor. Co-immunoprecipitation analysis of NirA-KapK interactions and subcellular localization studies of NirA mutants lacking different parts of the protein provided evidence that Met169 oxidation leads to a change in NirA conformation. Based on these results we propose that in the presence of nitrate the activation domain is exposed, but the NES is masked by a central portion of the protein (termed nitrate responsive domain, NiRD), thus restricting active NirA molecules to the nucleus. In the absence of nitrate, Met169 in the NES is oxidized by an FmoB-dependent process leading to loss of protection by the NiRD, NES exposure, and relocation of the inactive NirA to the cytosol.

## Author Summary

Nitrate serves as a major source of nitrogen nutrition for plants, algae and fungi, but the molecular details of how the nitrate signal is transduced to transcription factors regulating the expression of the nitrate assimilation genes are not known. To identify possible signaling mechanisms, we analyzed post-translational modifications in the pathway-specific activator NirA by mass spectrometry and found that NirA activity correlates with the oxidation status of a conserved methionine (Met169) in the regulatory nuclear export sequence (NES). This oxidation-reduction switch influences the overall conformation of the protein, which defines whether the NES is exposed or blocked. Site-directed mutagenesis and a forward-genetic suppressor screen identified two domains of NirA, which are regulating NES accessibility, subcellular distribution and the transcriptional activity of NirA. Our data for the first time establish a link between nitrate signaling and the redox status of the cell.

## Introduction

Nitrate is an important nitrogen source for fungi in natural environments. Most species of this kingdom possess an efficient enzymatic and regulatory system that allows conversion of nitrate to nitrite and further to ammonium, which is then incorporated into amino acids and other metabolites [1,2]. Nitrate represents the major soluble nitrogen form in soils and, besides serving as nutrient, also influences plant development [3–5], virulence of phytopathogenic fungi [6,7] and the production of fungal secondary metabolites [8,9]. Thus, elucidation of the molecular mechanisms underlying nitrate signalling in *A*. *nidulans* may serve as a model for other nitrate assimilating eukaryotes such as algae and plants. Marchive and colleagues [10] have shown that NLP7, the *Arabidopsis thaliana* nitrate-responsive transcription factor shuttles between the cytosol and the nucleus in response to nitrate availability in a similar way to NirA in *A*. *nidulans*.

Activation of the nitrate-specific transcription factor NirA in the ascomycete *Aspergillus nidulans* is a process which involves both nuclear retention of NirA and its conversion to a functional activator [11]. We previously found that intracellular nitrate or nitrite leads to disruption of the interaction between the nuclear export sequence (NES) of NirA and the specific exportin KapK, the CRM1 homologue in *A*. *nidulans* [12–16]. As a result NirA accumulates in the nucleus within less than a minute after the addition of nitrate (see S1 Video), and is subsequently able to bind to the UAS (upstream activating sequences) of genes involved in nitrate assimilation [17]. NirA target genes are only activated when nitrate is present and, at the same time, the intracellular concentration of glutamine, a crucial intermediate in nitrogen assimilation, is low [18]. NirA acts synergistically with the GATA-factor and glutamine sensor AreA to recruit chromatin acetylation and nucleosome remodelling activities [19–22]. NirA-AreA synergism leads to a rapid transcriptional activation of around 100 genes, among them those required for nitrate reduction and incorporation of the resulting ammonium into glutamate and glutamine. Upstream of these genes NirA and AreA binding sites are present. Genes involved in nitric oxide metabolism are also induced by nitrate but, interestingly, this process does only require NirA, but not AreA [18].

Our previous work established that nuclear accumulation, resulting from leptomycin B (LMB)-mediated inactivation of KapK, is not sufficient to activate NirA [11]. Thus, nitrate-induced activation of NirA involves at least two steps, i.e. release of KapK interaction resulting in nuclear accumulation and acquisition of transcriptional activation competence. In the *nirA* ^c^1 allele glycine 167 within the NES of NirA is replaced by a valine (G167V). This mutation not only leads to permanent, nitrate-independent NirA nuclear retention but also to constitutive binding to its DNA target-sequences and transcriptional activation of the cognate regulated genes. Conversely, replacement of consensus leucines in the NES required for KapK interaction also leads to constitutive nuclear accumulation but the protein is not functional [11]. These findings suggest that the NES region of NirA not only determines the subcellular distribution of the transcription factor but also its ability to act as activator protein. The rapid response of NirA nuclear accumulation to nitrate induction prompted us to investigate post-translational modifications (PTMs) as possible regulatory switches between cytosolic-inactive and nuclear-active NirA. To identify possible PTMs associated with this activation-inactivation cycle we affinity-purified NirA from non-induced and short-term (3 minutes) nitrate-induced cells and analysed the resulting proteins by mass spectrometry (MS). In this article we report that the oxidation status of a conserved methionine within the NirA NES is correlated with the subcellular distribution and transcriptional competence of the protein. We investigated the cognate oxido-reduction switch and based on genetic and biochemical data, we propose a role of an hitherto uncharacterised central domain of NirA which, interacting with the C-terminal activation domain, determines the accessibility of the NES in response to the oxido-reduction switch.

## Results

### Genetic constructs used in this work

The constitutive expression of the *nirA* gene is too low to allow biochemical analyses and cell localisation studies using GFP fusions. In previous work, expression was driven by constitutive (*gpdA*) or inducible ERE or *alcA* promoters [11,17]. Overexpression does not alter the response of NirA to regulatory signals [22] and thus we used these constructs in the work presented here. Western blots of the different NirA-GFP (expressed from *gpdA* or ERE promoters) or FLAG-NirA (expressed from the *alcA* promoter) constructs are shown in S5A and S5B Fig. Biochemical work was carried out with FLAG-tagged NirA driven by the *alcA* promoter under inducing (0.2% fructose plus EMK, a gratuitous inducer; see Materials and Methods) or derepressed conditions (0.2% fructose), allowing modulation of *nirA* expression. The latter was checked by both Northern (S3 Fig) and Western blots (S5B Fig).

### Met169 is present as its sulfoxide in the absence of nitrate

The NirA NES includes a highly conserved methionine (M169). This residue is present in 57 out of 68 known or predicted NirA orthologs, the only substitution detected being isoleucine, a residue of similar length and hydrophobicity, but not containing an oxidizable sulphur atom.

An extended alignment report can be found in B-Link BLAST results for NirA orthologs following http://www.ncbi.nlm.nih.gov/protein/259489709 (note: *A*. *oryzae* protein XP001824047 appears in this alignment based on the conserved binuclear Zn cluster domain but it is not a NirA orthologue). The conservation of M169 in the export sequences of NirA orthologues and the fact that the G167V exchange in NirA^c^1 leads to permanently nuclear and active NirA [11] suggests that the region bearing the NES significantly contributes to the regulatory characteristics and nitrate-responsiveness of NirA.

**Fig 1:**
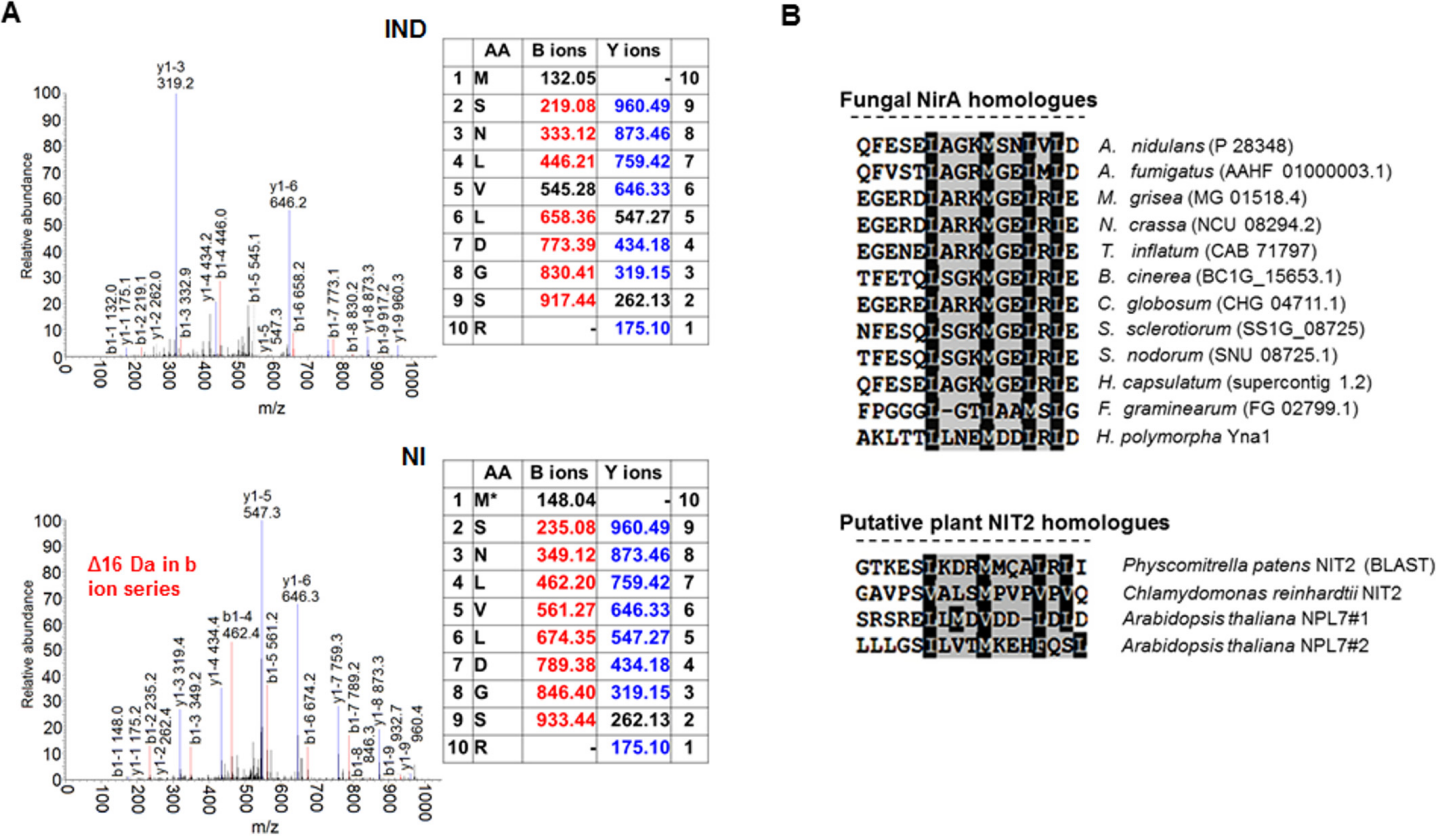
A conserved methionine in the NES of NirA is oxidized in the absence of nitrate. **(A)** Example of an MS/MS spectrum of NirA NES peptides derived from wild-type FLAG-NirA purified from cells grown under inducing (IND) and non-inducing (NI) conditions. For both conditions, cells were initially grown under NI conditions on 3 mM arginine for 14 hours and for induction, 10 mM NO_3_^-^ was added to a subset of cultures and incubation proceeded for 5 minutes prior to harvesting. The difference between methionine sulfoxide (M^ox^) and methionine (M) is indicated by the 16 Da shift of the B ion series in the MS/MS spectra. An overview of MS/MS data obtained from PTM analyses of NirA-NES methionine 169 (Met169/M^ox^169) in the wild type and different mutant strains is given in Table 1. Detailed MS/MS spectra are shown in S6 Fig AA, amino acids. **(B)** Alignment of known or putative NES sequences comprising methionine residues in their motifs. NirA homologous genes from fungi as well as plant proteins with known or proposed function in the nitrate response were selected. Fungal species harbouring NirA homologues are aligned as follows: *Aspergillus nidulans*, *Aspergillus fumigatus*, *Magnaporthe grisea*, *Neurospora crassa*, *Tolypocladium inflatum*, *Botrytis cinerea*, *Chaetomium globosum*, *Sclerotinia sclerotiorum*, *Stagonospora nodorum*, *Histoplasma capsulatum*, *Fusarium graminearum*, *Hansenula polymorpha*.

We analysed FLAG-tagged NirA obtained by DNA affinity-purification from cells grown on non-inducing (NI, 3 mM arginine) or nitrate inducing (IND, 10mM NaNO_3_) conditions by tandem mass spectrometry. In the absence of NO_3_^-^, the NES of NirA is modified by oxidation of the conserved methionine (Met169) to methionine sulfoxide (Met^ox^169). When cells were exposed to nitrate for five minutes Met^ox^169 could not be detected any longer (Fig 1 and Table 1). This rapid response of *A*. *nidulans* cells to the inducer coincides with the swift nuclear accumulation of NirA (S1 Video). No Met169 oxidation could be detected in the NirA^c^1 protein **(** Table 1 and S6 Fig). These MS results strongly suggested a functional link between the oxidation status of M169 and intracellular localisation of NirA.

**Table 1:** Overview of correlations between NES sequences, oxidation status of M169 (red/ox), NirA-GFP subcellular localization and NirA transcriptional activity in different genetic backgrounds of strains grown under non-inducing or inducing conditions.

Genetic background and NES composition	non-induced (arginine)			induced (nitrate)		
	red/ox	localization	activity	red/ox	localization	activity
WT—M169	M^(ox)^	cytosolic	-	M^(red)^	nuclear	+
WT—M169A	n.a.	nuclear	+	n.a.	nuclear	+
WT—M169I	n.a.	cytosolic	-	n.a.	nuclear	+/-
WT—G167V (*nirA* ^c^1)	M^(red)^	nuclear	+	M^(red)^	nuclear	+
WT—L172A/L174A	n.d.	nuclear	-	n.d.	nuclear	-
*fmoA*Δ - M169	M^(ox)^	cytosolic	-	M^(red)^	nuclear	+
*fmoB*Δ - M169	M^(red)^	nuclear	-	M^(red)^	nuclear	+
*msrAB*ΔΔ - M169	M^(red/ox)^	cytosolic	-	M^(red/ox)^	nuclear	+
WT—M169—*nirA* ^ADΔ^	n.d.	nuclear	-	n.d.	nuclear	-
WT—M169 –*nirA* ^NiRDΔ^	n.d.	cytosolic	-	n.d.	cytosolic	+/-

(n.a., not applicable, n.d., not determined)

To exclude that methionine oxidation was an artefact occurring during extract preparation we included serum albumin in all samples before cell disruption, and checked the oxidation status of methionines. No significant oxidation of albumin methionine was detected demonstrating that the NirA NES Met169 oxidation isolated from NI cells condition was condition-specific. Absence of Met^ox^169 in induced wild type and non-induced *nirA* ^c^1 mutant cells provided further evidence that methionine oxidation did not occur spontaneously. Furthermore, in all MS experiments, which were performed at least in triplicate, only one oxidation status of Met169 (Met169 or Met^ox^169) in the NES peptide was reproducibly detected (Figs 1A and S6).

Methionine can form two diastereomeric sulfoxides which are individually reduced to methionine by MsrA (Met^ox^-S reductase) and MsrB (Met^ox^-R reductase), respectively [23,24]. In a previous work we studied this thioredoxin-dependent pathway in *A*. *nidulans* [25] and the availability of a double *mrsAΔmrsBΔ* and a *trxAΔ* (thioredoxin-deficient) strain allowed us to test directly if Met^ox^169 reduction of NirA depends on MsrA, MsrB or thioredoxin [25,26]. No significant requirement of these proteins for Met^ox^169 reduction or NirA activity was found (see Table 1 and S1 Fig). MS analysis of FLAG-NirA in the *mrsA*Δ*mrsB*Δ strain revealed a mixed pattern of Met169 and Met^ox^169 under both NI and IND conditions and we noticed that in these proteins also other methionines (e.g. M719 in the NirA C-terminus) appear in the sulfoxide form. Moreover, the thioredoxin-deficient *trxAΔ* strain showed no reduction in NirA activity as tested by growth tests [25] and *niiA* transcription in the deletion strain (S1C Fig). These data indicate that absence of MsrA and MsrB leads to non-specific protein damage [25] but Met^ox^169 reduction still occurs. The partial oxidative damage of NirA could explain the slightly reduced transcriptional activation capacity of NirA found in the *msrA msrB* double deletion strain (S1B Fig). Because FLAG-NirA carries reduced M169 in the *msrA msrB* double deletion background it remains to be determined which activities would be required for the very rapid NirA-NES Met^ox^169 reduction associated with activation of NirA by nitrate.

**Fig 2:**
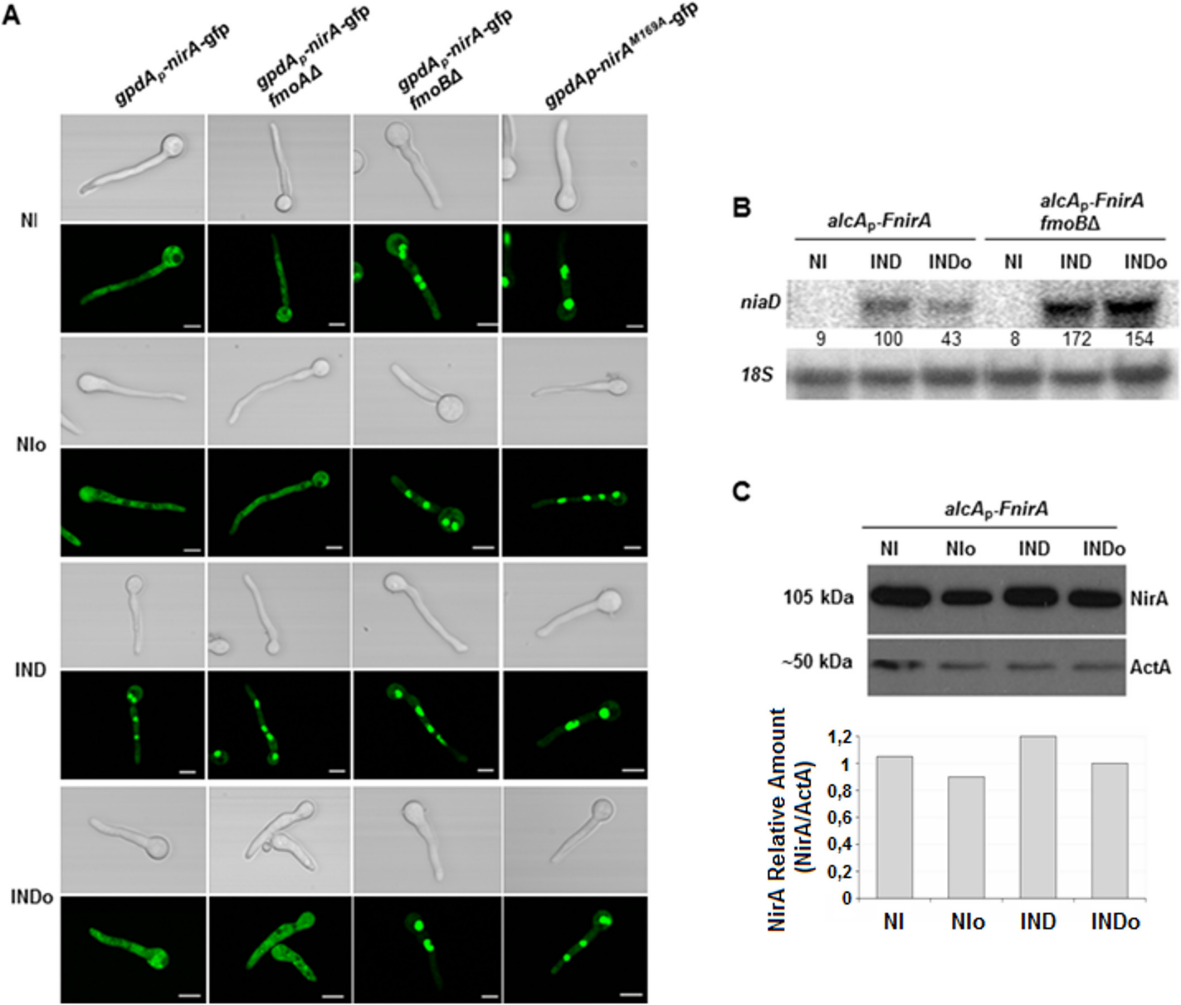
Methionine oxidation in the NirA-NES defines subcellular localization and activity. **(A)** NirA-GFP sub-cellular localization in different strains under non-induced (NI, 3 mM arginine), non-induced plus n-octylamine (NIo, 3 mM arginine plus 10 mM n-octylamine), induced (IND, 10 mM nitrate) and induced plus n-octylamine (INDo, 10 mM nitrate plus 10 mM n-octylamine) conditions. Pictures were taken two minutes after addition of the respective compounds. All strains harbour the Leptomycin B (LMB) sensitive allele *kapK*1. Leptomycin B sensitivity enables testing if loss of nuclear accumulation is related to/dependent on exportin (KapK) function. Except strain *kapK*1 NirA^M169A^, which carries a Met to Ala substitution within the NES sequence, all strains carry a NirA-GFP wild type construct. Scale bars refer to 5 μm. **(B)** Effect of the *fmoB* deletion on *niaD* transcription. Strains were grown under standard conditions and transferred for 20 minutes to the media as indicated and described in panel A. Signal intensities were calculated with ImageQuant (Molecular Dynamics) and *niaD* expression was normalized to *18S* rRNA. **(C)** Effect of nitrogen source and n-octylamine on NirA protein stability. Whole cell extracts of a FLAG-tagged NirA strain were analysed by Western blot. The strain was grown for 16 hours under non induced conditions with 0.2% fructose and 3 mM arginine as nitrogen source; NI. The cultures where subsequently treated with 2 mM n-octylamine, NIo; 10 mM nitrate, IND; and 10 mM nitrate plus 2 mM n-octylamine, INDo. Specific signals were normalized against actin (ActA).

As methionine oxidation could generally affect NirA protein stability under non-induced conditions, we tested the amounts of FLAG-NirA in crude extracts obtained from arginine-grown (NI) or NO_3_^-^grown (IND) cells. We found that NirA levels were basically equal under both conditions and this result indicates that overall stability of NirA is not influenced by the Met169 oxidation state (Fig 2C).

### Met^ox^169 formation and NirA nuclear export depends on FmoB, a flavin-containing monooxygenase

**Fig 3:**
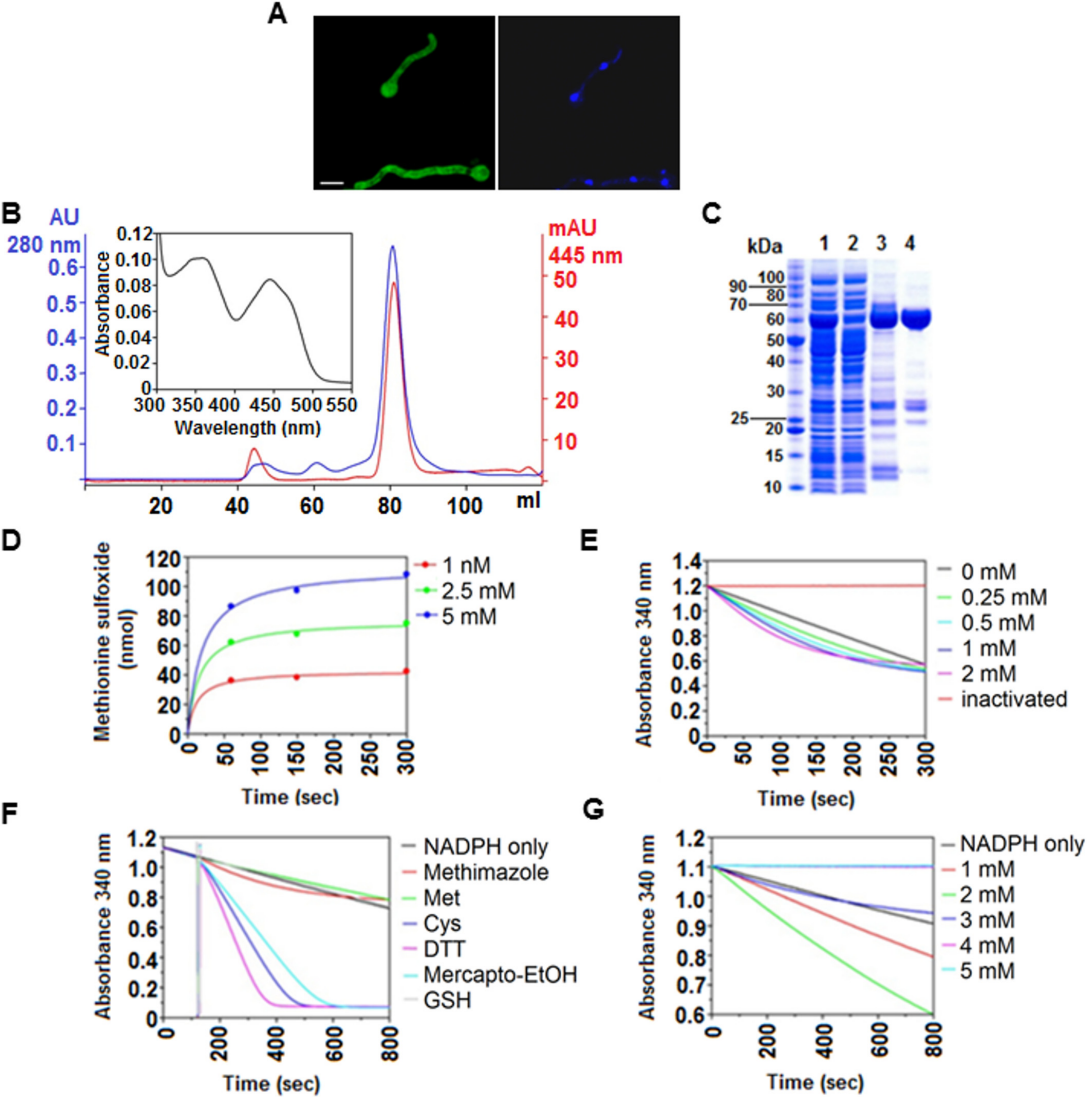
*In vivo* and *in vitro* characteristics of FmoB. **(A)** Sub-cellular localization of FmoB as a GFP fusion protein. FmoB::GFP localizes both in the cytoplasm and in the nuclei. Nuclei were visualized by DAPI staining. Cells were grown for 16 h in AMM with 3 mM arginine as a sole nitrogen source. Scale bar refers to 5 μm. **(B)** Preparative size exclusion chromatography elution profile of purified recombinant FmoB. An UV-visible absorbance spectra of FAD saturated FmoB is shown as inset. **(C)** SDS-PAGE analysis of FmoB purification steps ([1] HIS-Select load; [2] HIS-Select flow through; [3] HIS-Select pool; [4] size exclusion chromatography pool). The FmoB band corresponds to the molecular weight of ~ 60 kDa. **(D)** HPLC monitoring of L-methionine sulfoxide formation by FmoB. FmoB was incubated with indicated L-methionine concentrations for 60, 150 and 300 seconds and the formation of L-methionine sulfoxide was quantitatively analyzed by HPLC. Detected amounts (nmol) of L-methionine sulfoxide are displayed over the time and data points were fitted by the hyperbolic function y = ax/(b+x) using SigmaPlot 10. On the left panel, the concentrations of free methionine and the corresponding colours are indicated. **(E)** FmoB activity assay with methimazole. FmoB was incubated with indicated concentrations of methimazole as described in Supplementary Materials and Methods. Absolute values of change in NADPH absorption at 340 nm were plotted versus time. NADPH oxidase activity of the enzyme in the absence of substrate and heat inactivated FmoB in the reaction are shown as controls. There is only a slight increase in NADPH consumption when methimazole concentrations raise from 0,25 mM to 0.5 mM. On the left side of the graph, methimazole concentrations and the corresponding colours are indicated. **(F)** FmoB activity assays with different substrates. NADPH levels are expressed as absorbance (OD_340nm_) and are plotted against time. All samples received 2 μM FmoB and 200 μM NADPH. After 120 seconds pre-incubation time, samples received additionally 2 mM of putative substrates and kinetics of OD_340nm_ reduction indicates enzyme activities on the tested substrates. Cysteine, DTT and β-mercaptoethanol are apparently preferred substrates for FmoB under these conditions. **(G)** FmoB NADPH-oxidase activity is enhanced by n-octylamine. NADPH levels are expressed as absorbance (OD_340nm_) and are plotted against time. All samples received 0.5 μM FmoB and 200 μM NADPH and in addition indicated concentrations of n-octylamine. A specific substrate was omitted from this assay. Stimulation of FmoB activity apparently is optimal at concentrations between 1 mM and 2 mM, higher concentrations apparently inhibit the enzyme activity in these conditions.

Flavin-containing monooxygenases (FMOs) have been shown to oxidize peptide-bound methionines [27]. In the *A*. *nidulans* genome at least seven putative FMO-encoding genes were identified. These enzymes contain FAD and NADP binding sites and a FMO signature (S2 Fig). Based on these searches and on *A*. *nidulans* nomenclature we designated the putative enzyme most similar to *S*. *cerevisiae* FMO1 as FmoA (gene number ANID_08206.1) whereas the protein showing highest similarity to human FMO3 was designated FmoB (ANID_04110.1). We deleted both *fmoA* and *fmoB* genes and subsequently analysed NirA in the deletion strains. Met169 status and subcellular localization of NirA-GFP were unchanged in *fmoAΔ* cells but Met^ox^169 was not detected any longer in the *fmoBΔ* strain under any conditions (Table 1 and S6 Fig). We subsequently tested subcellular localization of FmoB by fusing *fmoB* to *gfp*. Unfortunately, this fusion, expressed from the weak homologous *fmoB* promoter, did not result in clear GFP labelling of cellular compartments and we thus expressed the GFP-tagged version of FmoB from the strong *gpdA* promoter. The resulting fusion protein was found to localize all over the cell without particular accumulation in any compartment (Fig 3A). Confocal microscopy of NirA-GFP however, showed that the transcription factor was constitutively nuclear in the *fmoBΔ* background (Fig 2A). This result is consistent with M169 oxidation in the NES under NI conditions having a specific role for NirA localization and function. However, despite the constitutive nuclear localisation of NirA, the *fmoBΔ* background does not lead to constitutive expression of NirA-dependent genes (Fig 2B). Induction by nitrate, on the other hand, occurred as in the wild type and may even be slightly enhanced (+70%) in the *fmoB* mutant (Fig 2B). These results confirm that the oxidation status of NirA-NES is determined specifically by FmoB, one out of seven flavin-containing monooxygenases predicted in the *A*. *nidulans* genome. These data also confirm that the NES oxidation status controls the nuclear-cytosolic shuttling process but not necessarily the transcriptional activity of NirA.

### FmoB can oxidize free methionine and cysteine but does not oxidise Met169 in a synthetic NES peptide

We next determined the NADPH-dependent enzymatic properties of FmoB and expressed the complete *fmoB* gene in *E*. *coli* (Fig 3B and 3C). In the absence of any substrate, FmoB displays a low NADPH-oxidase activity leading to gradual decrease in NADPH levels of roughly 30% over 12 minutes. Our assays and subsequent HPLC analysis demonstrated that recombinant FmoB is a functional methionine oxidase as it is able to convert free methionine to the sulfoxide form (Met^ox^). However, the rate appears to be low since only 100 nmoles Met^ox^ were formed during 5 minutes at a concentration of 5 mM free methionine (Fig 3D). The same low activity was observed with methimazole, which is another previously characterized FMO substrate [28]. NADPH oxidation in the presence of methimazole was hardly increased over the NADPH consumption background without substrate and increasing the amounts of substrates from 0.25 mM to 2 mM only marginally increased the NADPH consumption (Fig 3E). In contrast, other thiols such as free cysteine, dithiothreitol (DTT) or β-mercaptoethanol were favourable substrates for FmoB under our experimental conditions but, surprisingly, glutathione with its peptide-bound cysteine was not (Fig 3F). We next tested if recombinant FmoB is able to oxidize Met169 in a synthetic peptide carrying the NirA-NES. We used an 18 amino acid peptide containing the NirA-NES (DQFESELAGK**M**SNLVLDG) as substrate but could not detect any enzyme-dependent NADPH oxidation. These biochemical data suggest that FmoB is either not a peptide methionine oxidase or that it requires additional NirA sequences to recognize the NES and to oxidize Met169 in NirA. Alternatively or additionally, FmoB function may diminish the pool of reduced aliphatic thiols (such as free cysteines) and thereby indirectly promote Met169 oxidation. Taken together, our biochemical and genetic characterization suggests that FmoB is a functional thiol oxidase directly or indirectly required for Met169 oxidation and relocation of NirA to the cytosol.

### FmoB is involved in forcing NirA nuclear exclusion in nitrate-induced cells

**Fig 4:**
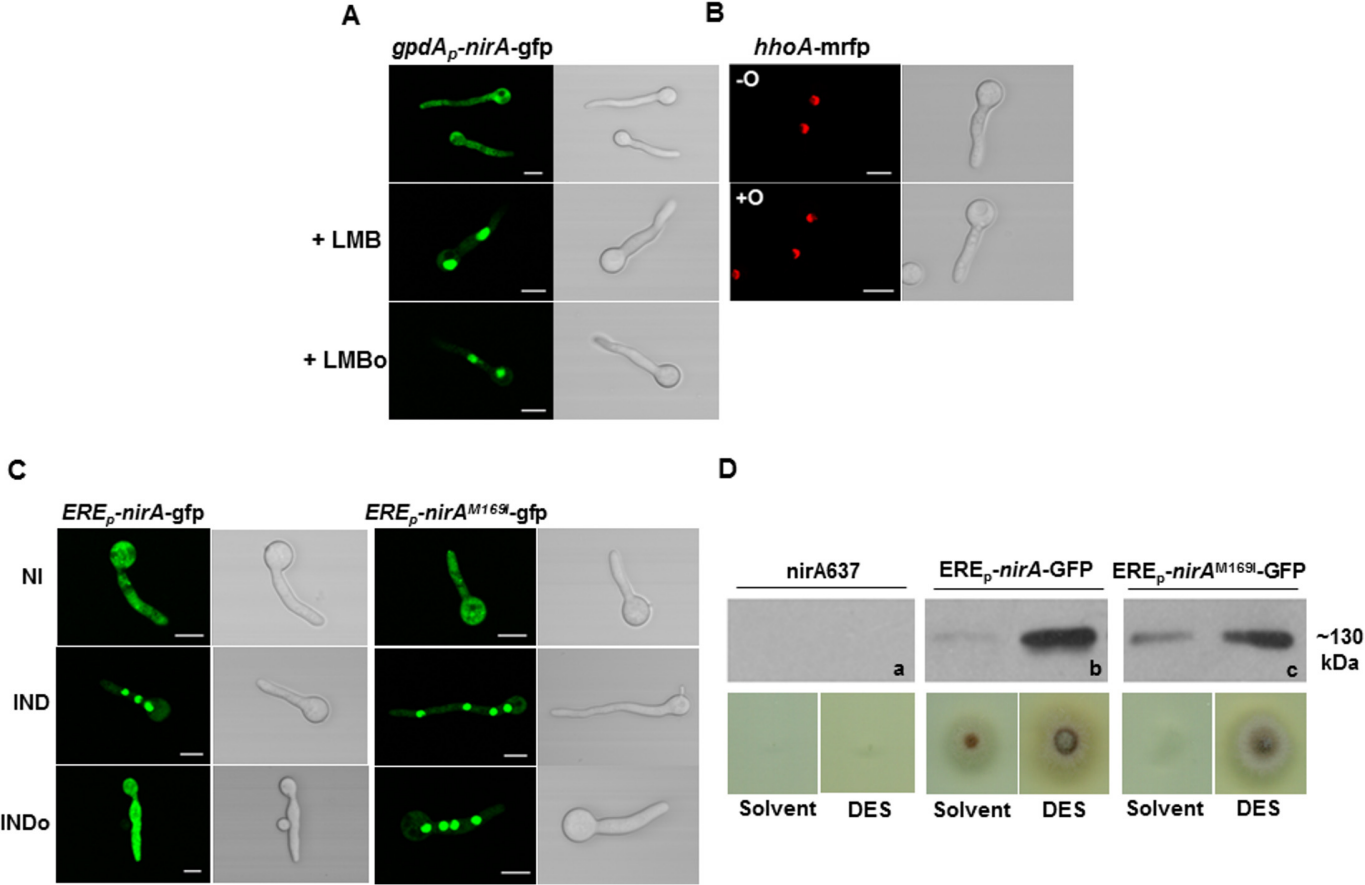
N-octylamine treatment does not influence NirA subcellular localization in KapK/CRM1-inactivated cells. **(A)** The *KapK*1 strain was incubated with 10 ng/ml Leptomycin B (+LMB) for 30 minutes and subsequently treated with 10 mM n-octylamine (+LMBo). **(B)** The control strain expressing histone H1 tagged with RFP (*hhoA*-*mrfp*) was used to verify that n-octylamine has no damaging effect on the subcellular localization of nuclear proteins.-O, in the absence of n-octylamine; +O, in the presence of 10 mM n-octylamine. Cells were grown 16 h on GMM with arginine 3 mM as nitrogen source. **(C)** Comparison of NirA sub-cellular localization between the control strain and the *ERE*_*p*_ *-nirA* ^*M169I*^ *-gfp* strain under non inducing (NI, arginine 3 mM), inducing (IND, nitrate 10 mM, 2 minutes), inducing plus n-octylamine (INDo, nitrate 10 mM, 10 mM n-octylamine, added after 2 minutes of nitrate induction) conditions. Scale bars refer to 5 μm. **(D)** Growth test of *nirA637*, *ERE*_*p*_ *-nirA*-*gfp* and *ERE*_*p*_ *-nirA* ^*M169I*^-*gfp* strains in the presence of DES (diethylsilbestrol), inducer of *ERE* promoter on nitrate. Western panels (a, b, c) show the amount of NirA-GFP fusion protein (~130 kDa) in each strain tested for growth.

N-octylamine (abbreviated NOC in the text or “o” in figure labellings) is a compound which accelerates some FMO-mediated processes [29]. We thus tested whether purified FmoB responds to NOC. Addition of 1mM or 2 mM NOC to the *in vitro* reaction mixture noticeably increases the NADPH-oxidase activity of the enzyme, whereas higher concentrations were inhibitory (Fig 3G). When we tested NOC effects *in vivo* we found a striking effect. NirA-GFP was rapidly exported from the nucleus after addition of 10 mM NOC to NO_3_^-^ induced wild type cells and this NOC-triggered exclusion did not occur in *fmoBΔ* cells (Fig 2A). To exclude the possibility that the observed cytosolic staining was simply a consequence of NirA degradation and subsequent random distribution of GFP within the cells we tested NirA stability after NOC treatment by Western blotting. Extracts prepared under denaturing conditions (TCA preparation to avoid non-specific degradation during processing) from FLAG-NirA cells grown under NI and IND condition in the presence and absence of NOC showed that NirA was not degraded during treatment with this chemical (Fig 2C). We also wanted to exclude the possibility that NOC treatment leads to a defect in nuclear integrity and subsequent random GFP distribution. The results demonstrate that this is not the case as NOC did not lead to cytoplasmic localization of NirA-GFP in a *fmoBΔ* strain (Fig 2A), nor did it lead to loss of nuclear positioning of a histone H1-mRFP fusion protein (strain *hhoA-mrfp*, Fig 4B).

### The KapK exportin and Met169 in the NirA NES are required for the FmoB-triggered nuclear exclusion

The above results suggest that the FmoB-triggered nuclear exclusion of NirA depends on active export process. In this case, the loss of NirA nuclear accumulation under IND conditions should be KapK dependent. To verify this, we treated the induced cells with NOC and simultaneously inactivated KapK1 with leptomycin B (LMB). Under these treatments the effect of NOC was lost and NirA-GFP remained nuclear (Fig 4A). This finding confirmed that the FmoB-dependent nuclear exclusion of NirA-GFP triggered by NOC is an active process, which depends on the function of the exportin KapK. As expected, the loss of NirA nuclear accumulation negatively impacted on NirA transcriptional activity. In the wild type background NOC treatment led to a significantly lower level of the *niaD* transcript (ca. 60% reduction) whereas reduction of transcriptional activity is only marginal (ca. 10% reduction) in NOC-treated *fmoBΔ* cells (Fig 2B).

Although the above results suggest that the NOC-triggered effect is related to Met169 oxidation we wanted to directly determine if NOC treatment leads to Met^ox^169 formation and the resulting NirA cytoplasmic localization and loss of transcriptional activating properties. To this end we tried to affinity purify FLAG-NirA from NOC-treated cells but, we were not able to obtain sufficient amounts of FLAG-NirA for mass spectrometry. As an alternative to test the involvement of Met169 oxidation in the export process, we mutated M169 to isoleucine (NirA^M169I^) which is functionally and sterically similar but cannot be oxidized. Fig 4C shows that NOC treatment (INDo) of this strain did not lead to NirA^M169I^–GFP nuclear exclusion. This result establishes that Met169 is the only amino acid targeted by the FmoB-dependent process that leads to nuclear exclusion of NirA under nitrate induced conditions in the presence of NOC.

### Met169 substitution by isoleucine results in a partial loss-of-function phenotype

Given the vital role Met169 plays in NirA regulation, we tested if isoleucine, a sterically and biochemically similar residue which cannot be oxidized, could mimic reduced methionine in active NirA. We thus replaced *nirA* ^+^ with *nirA* ^M169I^ and tested the subcellular distribution of NirA^M169I^-GFP under conventional growth conditions (in the absence of NOC). We found that NirA^M169I^-GFP subcellular distribution was indistinguishable from wild type NirA-GFP, i.e. cytosolic in NI and nuclear in IND conditions (Fig 4C) If, as hypothesized earlier, Met^ox^169 has a regulatory role, some phenotype (either a gain-of-function or a loss-of-.function phenotype) should become apparent. To test NirA^M169I^ function we constructed a strain in which the *nirA* ^M169I^ gene was driven by the estrogen-responsive promoter which is a hybrid sequence composed of roughly 100 bp of the *nirA* endogenous promoter fused to three estrogen-responsive elements and a 93 bp random sequence (for details on promoter composition see [30]). This [(3xERE)-RS-*nirA*] construct was chosen to be able to study the function of the mutated NirA^M169I^ variant at near physiological levels (using part of the *nirA* promoter) but with the possibility to increase the protein concentration by ERE-mediated estrogen induction. Fig 4D shows the Western analysis testing protein levels of the NirA-GFP wild type (ERE_p_-*nirA-gfp*) and NirA^M169I^-GFP variant (ERE_p_-*nirA* ^M169I^ *-gfp*). In the absence of estrogen the proteins were barely detectable but were highly abundant in the presence of estrogen (DES).

At levels comparable with those obtained with the *nirA* physiological promoter (in the absence of DES) the NirA^M169I^ substitution results in a clear growth phenotype on nitrate as sole nitrogen source, a phenotype which is not seen at higher expression levels of NirA (in the presence of DES), implying that that the M169I substitution is a hypomorphic mutation impairing but not completely abolishing NirA function. This effect is still evident in a strain expressing NirA^M169I^ from the slightly stronger *alcA* promoter (S4B Fig, de-repressed with 0.2% fructose), as we found a roughly 50% reduction in NirA^M169I^ activity under nitrate-induced conditions (*alcA*_*p*_-*FnirA* ^M169I^) compared to the wild type control (*alcA*_*p*_-*FnirA*).

### Met169 substitution by alanine results in a NirA constitutive phenotype

We next exchanged M169 to alanine, a small and hydrophobic residue that does not occur in the NES of any known or predicted NirA orthologues. NirA^M169A^_–_GFP showed permanent, nitrate-independent nuclear accumulation and as for the NirA^M169I^-GFP mutation, NOC treatment was ineffective (Fig 2A, last panel). To test the activity of the protein a FLAG-tagged version of NirA^M169A^ was expressed at moderate levels from the de-repressed *alcA* promoter (see *nirA* expression levels of two independent *alcA*_*p*_ *-FnirA* ^M169A^ transformants and corresponding protein levels of one strain in S5 Fig). Notably, the M169A variant resulted in a gain-of-function phenotype as this NES variant was constitutively active and promoted inducer-independent transcription of the NirA target gene *niaD* (S3 and S5 Figs, lanes *alcA*_*p*_ *-FnirA* ^M169A^ **)**. Thus, a *nirA* ^M169A^ substitution showed the same phenotype as the constitutively active *nirA* ^c^1 allele (carrying a *nirA* ^G167V^ substitution) and together these data support the view that the NES not only dictates NirA subcellular distribution but also its transcriptional competence.

### NES accessibility and the interaction with KapK depends on the Met169 oxidation state

**Fig 5:**
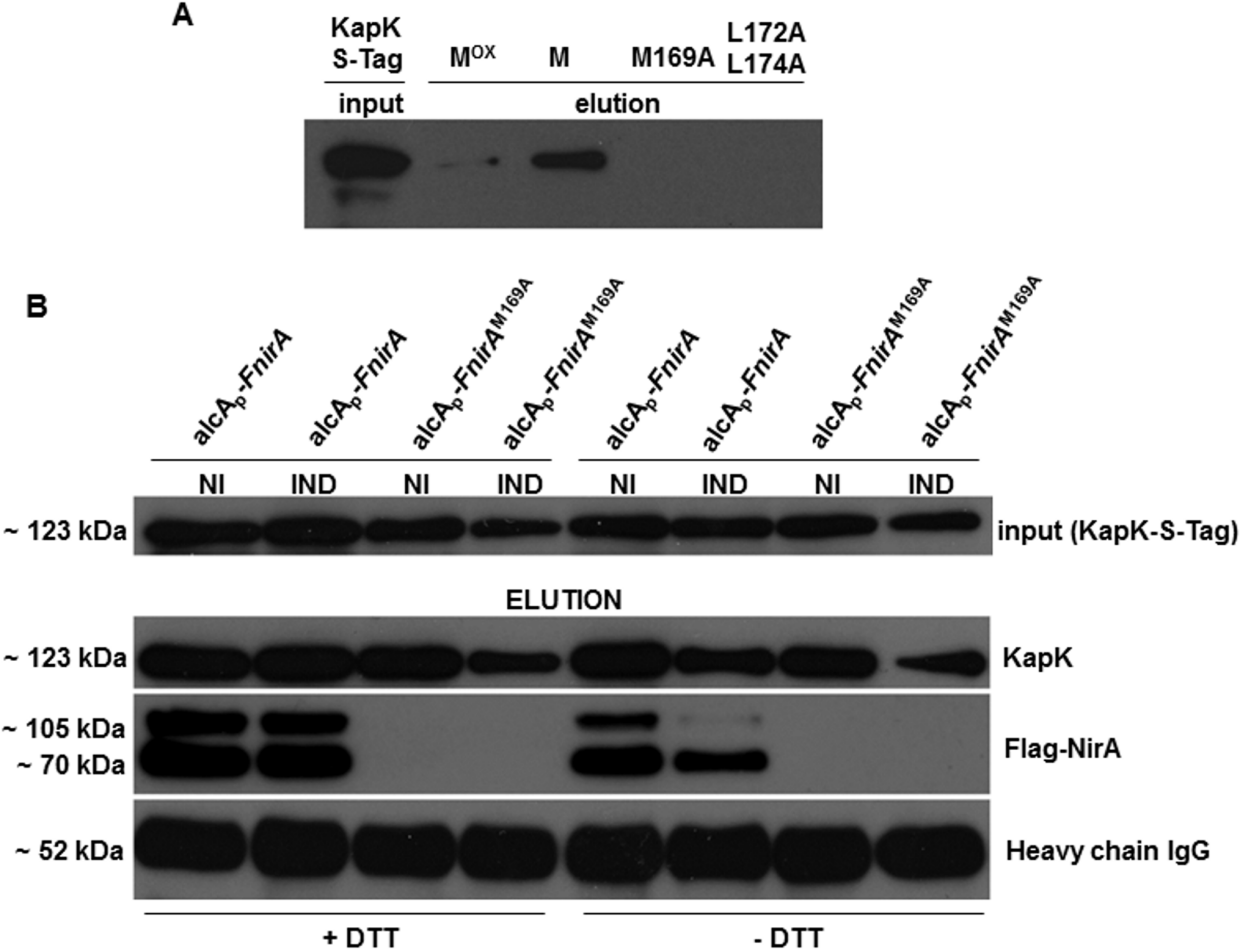
Interaction of KapK with the NirA-NES is sensitive to methionine oxidation. **(A)** Western blot using S-tag antibody shows *in vitro* interaction of KapK with synthetic NES peptide variants containing wild type or mutated amino acid sequences. M^ox^, NES peptide carrying methionine 169 sulfoxide; M, NES peptide carrying reduced methionine 169; M169A, NES peptide carrying a M169A substitution, L172A_L174A, NES peptide carrying a double L172A, L174A mutation. Signal densities reflect the interaction strengths between biotinylated NES peptides, immobilized on streptavidin sepharose, and KapK-Stag in non-induced cell-free extracts. **(B)** Western blot of co-IP experiments showing that *in vivo* NirA-KapK interaction is sensitive to the thiol redox status and to the presence of the C-terminal activation domain. Co-immunoprecipitation of cell-free extracts prepared from a strain co-expressing FLAG-NirA and KapK-S-tag. Extracts were prepared from cells grown under non-induced conditions (NI, 3 mM arginine) or induced conditions (IND, 10 mM NO_3_^-^). The extracts were prepared either in the presence (+), or absence (-) of 5 mM DTT. Input of pre-cleared cell-free extracts of all strains is shown in the upper panel. Elutions after the Co-IPs were separated on SDS-PAGE and equal loading of the co-IP resin with the S-tag capture Ab was verified by probing with the secondary Ab against the IgG heavy chain (~52 kDa) of the anti-S-tag antibody. A 70 kDa band of a proteolytically processed form of FLAG-NirA lacking around 30 kDa at the C-terminus is present.

We next investigated to which extent oxidation or the substitution of Met169 by other amino acids influences the interaction between the NES and KapK. Structural work established that the interaction of Crm1 (KapK homolog in *S*. *cerevisiae*) with nuclear export sequences requires hydrophobic residues [12,13]. Methionine with its hydrophobic side chain conforms to this rule but oxidation to its sulfoxide (Met^ox^) makes it more hydrophilic and polar [31]. It seems contradictory that, under conditions, in which NirA is effectively exported from the nucleus and NES-KapK interaction should be strong, oxidation of Met169 occurs, thus weakening precisely this interaction. We thus tested different NirA-NES synthetic peptides for *in vitro* interaction with S-tagged KapK present in protein extracts. These were prepared under native, non-denaturing conditions from KapK_S-tag cells grown on arginine medium (NI conditions). The 18 amino acids synthetic peptides spanning the NirA-NES (underlined; D^159^QFESELAGK**M**SNLVLDG^176^) comprised either the conserved M169 (bold) in non-oxidized (Met169) or oxidized (Met^ox^169) form or a M169A substitution. One additional peptide which carried two mutations (L172A/L174A) known to interrupt the NES-Kap interaction was also tested [11]. The biotinylated peptides were immobilized separately on streptavidin resin and incubated with cell-free extracts containing S-tag-KapK. After washing, the columns were eluted and the amount of S-tag KapK was determined by Western blotting with an anti-S-tag antibody (Fig 5A). In agreement with data above, we saw no interaction in the assay when the M169A or L172A/L174A peptides were used but a clear interaction with the M169 peptide (lane “M” in Fig 5A). When the NES peptide containing Met^ox^169 was tested the interaction with KapK was considerably weaker as documented by the much lower amount of KapK_S-tag captured compared to reduced Met169 (compare lanes “M” and “M^ox^” in Fig 5A). These *in vitro* binding assays confirm that direct interaction between KapK and the NES only occurs when the NES consensus is intact (i.e. hydrophobic residue of a specific length at position 169) and indicate that in fact reduced methionine in the NES results in a stronger interaction with KapK than oxidized methionine. This results pose the question of why, in conditions correlating with active export (Met169^ox^ under NI conditions), the interaction between the exportin and the NES is actually weaker than in conditions where the NES is presumably not functional *in vivo* (Met169 under IND conditions).

### Met^ox^169 indirectly determines NES-KapK interaction by affecting NES accessibility

The above paradoxical finding could be accounted for if Met^ox^169 –although weakening the KapK interaction,–had a regulatory function affecting protein structure and folding and thereby influencing either directly *via* domain exposure, or indirectly *via* protein-protein interactions, the accessibility of the NES to exportin. There are 12 cysteines in NirA, besides those involved in chelating the Zn^++^-ion in the bi-nuclear cluster. Querying the DiANNA disulphide prediction server (http://clavius.bc.edu/~clotelab/DiANNA/) with the NirA sequence omitting the cysteines in the DBD (query sequence residues 71 to 892) predicts four disulfide bonds whereas the same query submitted to DINOSOLVE (http://hpcr.cs.odu.edu/dinosolve/) predicts five disulfide bonds but also in this prediction, the cysteines potentially involved in disulfide formation partially overlap with the residues predicted by the DiANNA program. We tested this possibility by comparing the interaction of KapK with NirA in co-immunoprecipitation (coIP) experiments using extracts prepared from cells grown under NI (NirA export) or IND (no NirA export) conditions. To test if the NirA-KapK interaction is sensitive to disulphide bridge-mediated protein folding we performed the coIP experiments with two biochemically different setups, i.e. conditions, in which the natural folding of the protein is disrupted by addition of dithiothreitol (DTT) known to reduce disulfide bridges within or between proteins and under native conditions omitting DTT thus not changing any potential disulfide bonds within NirA.

To this aim we performed the coIP analysis in extracts of a strain producing both FLAG-tagged NirA (driven by the de-repressed *alcA* promoter, expression levels see S3 Fig) and S-tagged KapK (expressed from its native promoter). We also checked the coIP in a strain that carried the M169A substitution (FLAG-NirA^M169A^), which should not interact with KapK under any condition. Both strains were grown under standard non-inducing (NI, 3 mM arginine) and inducing (IND, 10 mM NO_3_^-^) conditions and the co-IP was carried out as detailed in Materials and Methods. In the absence of DTT (native folding), FLAG-NirA containing the wild type NES (lane *alcA*_p_-F*nirA*) co-eluted with KapK only under non-induced (NI) conditions and in this case a clear signal in the Western analysis was obtained (Fig 5B, right panels labelled [–DTT]). In contrast, under induced conditions (IND), only a very weak FLAG-NirA signal was detected. In extracts prepared from the strain carrying the M169A mutation (*alcA*_p_-F*nirA* ^M169A^) FLAG-NirA never co-precipitated with KapK. These pull-down assays confirmed the *in vitro* interaction data (Fig 5A) and demonstrated that *in vivo* the constitutive nuclear positioning of NirA^M169A^-GFP is due to the fact that the NES in NirA^M169A^ is not recognized by KapK.

When co-IP was carried out in extracts prepared under disulfide-reducing conditions (addition of 5mM DTT) which should unfold the native protein structure, FLAG-NirA permanently interacted with KapK-S-tag, regardless of whether the coIP was carried out with extracts from NI or IND cells (Fig 5B, left panels labelled +DTT). Thus, under IND conditions the NES must be masked by intramolecular interactions with are sensitive to DTT. Under NI conditions the NES seems to be permanently accessible to KapK. Interestingly, when the native protein structure was unfolded, the interaction between NirA and KapK is equally strong regardless of whether the NirA NES carries Metox169 (NI conditions) or Met169 (IND conditions). Thus the NES activity in the NirA protein depends on its global folding, rather than on the oxidation state of Met169. Under native protein folding conditions the NES of NirA (containing Metox169) is exposed only when the extracts have been prepared from NI cells but the NES containing reduced Met169 is not exposed or at least not accessible for KapK interaction when the extracts have been prepared from nitrate-grown (IND) cells.

### A C-terminal portion of NirA influences KapK-NES interaction and NirA subcellular localization

In the co-IP experiments we detected besides the expected Flag-NirA 105 kDa band a ∼70 kDa band. It is reasonable to suppose that this band results from a C-terminal truncation of N-terminal-FLAG-tagged-NirA. Whether this truncation occurred intracellularly or artefactually during extraction, this result shows that a C-terminally truncated NirA can bind to KapK irrespectively whether the extracts have been prepared from NI or IND cultures or whether the extracts have been treated with DTT or not. This faster migrating ~70 kDa band is most likely not an unspecific signal because it is not appearing in the extracts from the NirA^M169A^ strain. In other words, the deletion of the C-terminus has the same effect as unfolding the full-length protein by DTT. In both cases the NirA NES is able to interact with KapK in extracts prepared from nitrate-induced cells.

**Fig 6:**
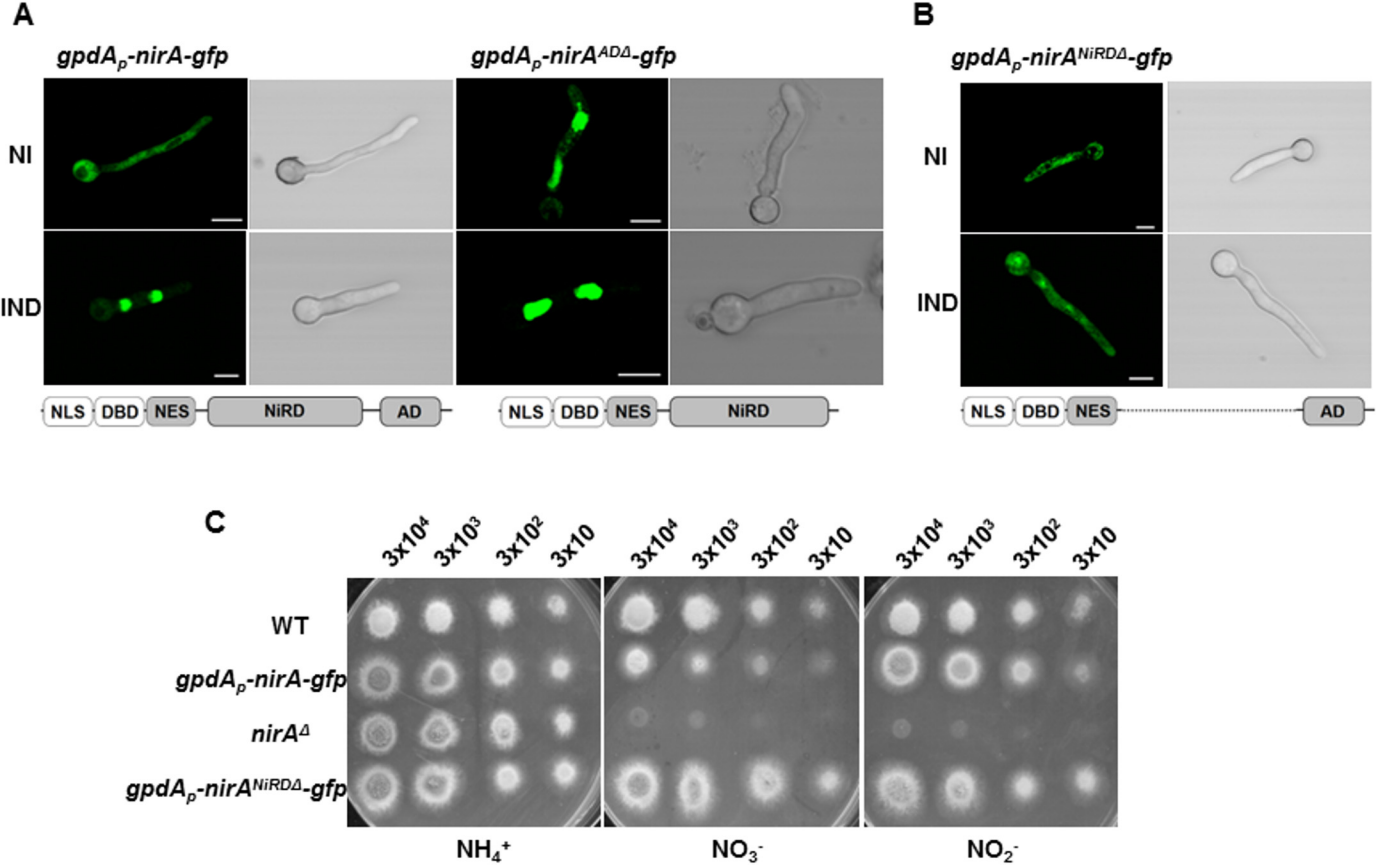
The C-terminus and a central portion of NirA participate in determining NES accessibility. **(A)** Lacking of the NirA activation domain (panel *kapK*1 NirA^-AD^) leads to permanent, nitrate-independent nuclear localization of a NirA-GFP fusion protein. Strain *kapK*1 served as wild type control. Fluorescence microscopy shows cells grown in GMM with 3 mM arginine as a sole nitrogen source (NI) and cells induced for 5 minutes by 10 mM nitrate (IND). This strain shows in the conditions tested a stronger GFP signal if compared to the other analysed strains. **(B)** The NirA-form lacking the central region cannot accumulate in the nucleus and is only moderately active. The expressed construct fused 364 amino acids of the N-terminal part (containing the nuclear localization sequence, DNA-binding domain and the NES) with the last 154 amino acids of the C-terminal AD region. The figure captures fluorescence microscopy pictures of the strain expressing NirA^NiRDΔ^-GFP under non-induced (NI, 3 mM arginine) or induced (IND, 10 mM nitrate) growth conditions. Scale bars refer to 5 μm. **(C)** Comparison between the growth on ammonium (NH_4_), nitrate (NO_3_^-^) and nitrite (NO_2_^-^) of *gpdA*_p_-*nirA* ^NiRDΔ^-*gfp* strain, the wild-type strain (WT) and two different strains harbouring respectively the *nirA* deletion (*nirA* ^Δ^) and the functional *nirA-gfp* expression driven by *gpdA* promoter (*gpdA*_*p*_ *-nirA-gfp*). The strain lacking NiRD domain of *nirA* shows growth on nitrate and nitrite comparable to that one on ammonium. The approximate number of inoculated spores is indicated.

To further investigate the role of the NirA C-terminus in the NES accessibility to KapK we constructed a *gpdA*_p_-NirA-GFP variant lacking 278 amino acids of the C-terminus (encompassing the previously defined 193 amino acids of the NirA activation domain, AD) and expressed this construct in *A*. *nidulans* (strain termed NirA^ADΔ^-GFP expressing amino acids 1–614 of NirA). If the C-terminal AD be functionally involved in masking the NES under IND conditions, a C-terminally truncated form should always expose the NES and never accumulate in the nucleus. Against our expectations, NirA^ADΔ^-GFP showed nuclear GFP staining constitutively, even when no nitrate was present in the medium (Fig 6A). Thus, the 278 amino acid domain of NirA is involved in two apparent functions. It is necessary for expression of genes regulated by NirA, as it includes the activation domain, but also includes a sequence or structure determinant that is necessary for NES exposure under NI conditions. While the proteolysed form always interacts with KapK (Fig 5), the *in vivo* behaviour of NirA^ADΔ^-GFP implies that it does not. This could be explained if the truncation resulting from proteolysis removed residues beyond the 278 C-terminus and this additional stretch was affected directly or indirectly the NES/KapK interaction.

### A central region of NirA is necessary to mask the NES

We thus wanted to characterize this region and deleted in the *nirA* gene the sequence coding for the central portion between the NES and the activation domain of NirA. This "minimal" version of NirA displayed a deletion from aa 230 to 737, thus fusing the N-terminal quarter of the protein (putative nuclear localization sequences, the DNA binding domain, and a stretch containing the NES) to the 193 amino acids of the AD thus removing all putative internal regulatory sequences (to be called nitrate regulatory domain, NiRD, and the strain lacking this region consequently NirA^NiRDΔ^). This construct, driven by the strong *gpdA* promoter and C-terminally fused to GFP was tested for subcellular distribution. Remarkably, the strict nuclear accumulation after nitrate induction is lost in this NirA variant and it is evenly distributed between nuclei and cytoplasm under both non-induced and induced conditions (Fig 6B). This demonstrates that not the AD but a central portion of NirA is required to mask the NES and most likely, the proteolytically processed form always interacting with KapK in the coIP experiments lacks an essential part of this regulatory domain of NirA. Growth tests performed with this strain showed that this “minimal NirA” is still sufficiently active to support growth on nitrate and nitrite as nitrogen sources, although overexpression of the construct may obscure some subtle differences in growth (Fig 6C).

### A forward-genetic screen pin-points a short stretch within the NiRD with putative regulatory functions

To narrow down the roughly 500 amino acid large region with regulatory functions and/or to identify extragenic factors mediating the NES-inhibitory effect of the NiRD we performed a screen for revertants of a conditional, cryo-sensitive mutations in *nirA*. Cryo-sensitive mutations are supposed to pin-point regions of protein-protein interaction, as they result in an increased energy requirement of activation for the assembly of specific structures [32]. We have selected (see Materials and Methods) a cryo-sensitive mutant unable to grow on nitrate at 25°C but able to grow at 37°C. We mapped the mutation to the *nirA* locus and sequencing of this allele identified a missense mutation resulting in a R347S substitution (see S2 Table for nucleotide changes of the individual mutations). R347 is strictly conserved among NirA homologues (see B-link results). We have selected revertants of this mutation, which all turned out to be intragenic suppressors. We identified ten different mutations which led to a suppression of the conditional *cs* phenotype (Table 1) and 7 out of these mapped in a very narrow region immediately upstream of the R347S *cs* mutation between amino acid 302 to 315 and, additionally, at positions 342 and 344. Bracketing the 302–315 region, basic residues were replaced by acidic ones (K302E, K315E) but within these boundaries the amino acid substitutions maintained or increased hydrophobicity (I305L, L306F, N308I, E310A, V312F). The V312F not only suppressed the original R347S mutation, but it resulted in constitutive gene expression of *niaD* and *niiA*.

## Discussion

To the best of our knowledge, this is the first report which demonstrates a regulatory role of Met oxidation on the function of a transcription factor. We have demonstrated a condition-specific oxidation of a conserved methionine in the NES of NirA which correlates with the relocation of the factor from the nucleus to the cytosol. *In vivo* data shows that M169 oxidation necessitates an active flavoprotein (FmoB) even though the cognate enzyme expressed in *E*.*coli* (rFmoB), which can catalyse cysteine, DTT and β-mercaptoethanol oxidation (Fig 3), failed to oxidise the Met of a synthetic NES peptide. Thus, FmoB could mediate Met169 oxidation in the NES directly or indirectly. The former would require NirA sequences outside the NES to be necessary for this FmoB specific activity. Such direct methionine oxidation has been shown only for MICALs (for Molecule Interacting with CasL), an animal protein involved in actin oxidation and de-polimerisation [33,34].

There are multiple possible indirect pathways *via* which FmoB could act to mediate NES oxidation. FMOs oxidize a wide range of compounds in microbes, plants and animals [33,34]. The *S*. *cerevisiae* FMO1p enzyme makes a major contribution to the pools of oxidized thiols and thus influences the cellular redox environment [35]. It could be proposed that FmoB in *A*. *nidulans* alters intracellular redox balance, which subsequently results in Met169 oxidation of the cytosolic and/or nuclear fraction of NirA. However, FmoB is not isofunctional to FMO1p. The substrate specificity of FMO1p is broader than FmoB and it is localised specifically in the endoplasmic reticulum [36]. Our experiments have demonstrated that, in the absence of substrate, FmoB is an NADPH oxidase releasing highly reactive hydrogen peroxide (H_2_O_2_), similar to the activity of human FMO isoforms [37], which by itself would alter the oxido-reduction state of the cell.

**Fig 7:**
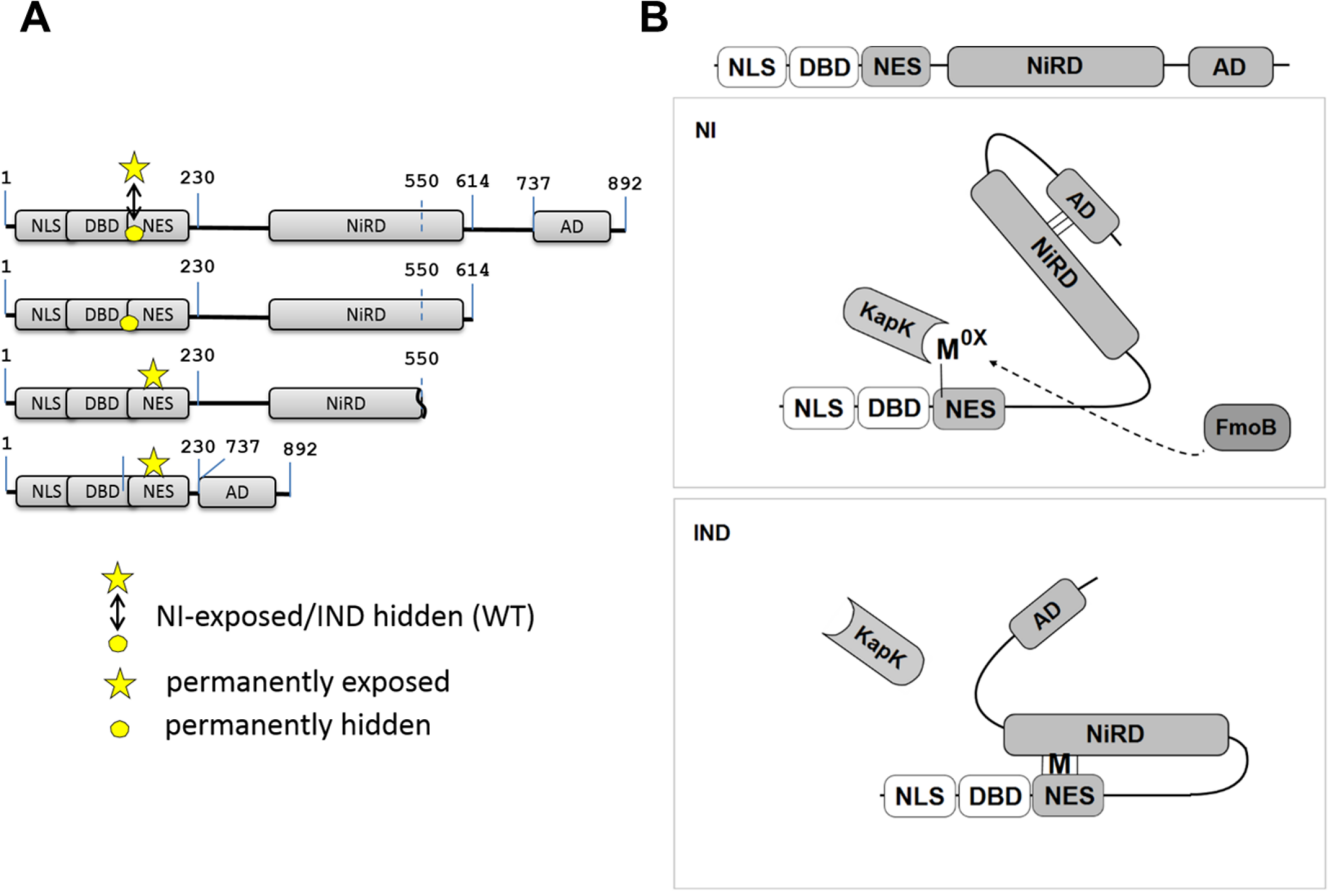
Model of NirA activity regulation. **(A)** Maps of NirA truncated forms highlighting the permanently exposed (indicated by the star) or permanently hidden (indicated by the circle) M169 on the NES. The digits indicate positions of residues in the NirA protein. In NirA-WT form, M169 is exposed or hidden in NI or IND condition, respectively and this is indicated by a double-headed arrow. **(B)** Under non-induced conditions (NI) the central nitrate regulatory domain (NiRD) interacts with the C-terminal activation domain (AD) and this interaction prevents the NiRD from masking the NES. Interaction with KapK is possible and the protein is continuously exported from the nucleus. The oxidized methionine (M^ox^) in the NES is proposed to stabilize this conformation. Details are described in the text. Under inducing conditions (IND), the presence of nitrate leads to a rapid accumulation of a reduced methionine in the NES and in this molecule the NiRD can efficiently interact with the reduced NES thereby masking it and liberating the activation domain. NLS, nuclear localization signal; DBD, binuclear Zn-cluster DNA-binding domain; NES, nuclear export signal; NiRD, nitrate-regulatory domain; AD, activation domain; KapK, nuclear exportin; FmoB, flavin-containing monooxygenase B; M^ox^, oxidized methionine; M, reduced methionine.

Structural studies of the human CRM1-snurportin1 places the LR-NES in a hydrophobic groove thus providing a structural basis for the hydrophobic NES consensus with intervening electronegative residues [38]. We have demonstrated the requirement of hydrophobic residues such as leucines L171/173 as well as of glycine G167 for NirA-NES function [11]. M169 can be replaced by isoleucine, but not by a small, less hydrophobic amino acid (alanine). The NirA NES contains oxidized Met169 under conditions in which it interacts efficiently with the KapK exportin. This is counter-intuitive as Met oxidation renders the NES more hydrophilic. This apparent contradiction could be resolved if the relevant parameter for the interaction of the NES and KapK was the exposure and availability of the NES, whether Met169 be oxidised or not. Immunoprecipitation (IP) and deletion analysis of NirA internal regions support this hypothesis. NirA in induced samples containing reduced Met169 can only be precipitated with KapK when the protein extract is treated with the strong thiol-reducing agent DTT (Fig 6B, left panels) known to reduce disulphide bonds [36]. This reducing treatment is presumably unfolding NirA and this may consequently expose the NES and allow KapK interaction in induced samples. Secondly, a partially degraded, C-terminally truncated form of NirA (called NirA^trunc^, a peptide roughly spanning residues 1–550, see Fig 6B and graphical representation in Fig 7A) always co-precipitated with KapK irrespectively of unfolding by DTT treatment which implies that the C-terminal ~350 residues contain a region which blocks NES accessibility.

We thus hypothesised that Met169 oxidation acts indirectly by influencing NirA conformation. A NirA construct lacking a shorter part of the C-terminus (NirA^ADΔ^-GFP expressing a NirA peptide spanning residues 1–614, see Figs 6A and 7A) showed the opposite effect and *(in vivo*) never interacted with KapK indicating a permanent block of the NES. This latter result delimits the region which affects the accessibility of the NES.

We propose that a region between NirA residues of around 550 to 614 is involved in sequestering the NES and that the region from residues 615–982, comprising the activation domain (AD), negates the sequestering function of region 550–614. A version of NirA composed of only the N-terminal 230 residues (NLS, DBD and the NES) fused directly to the very C-terminal 155 residues (covering the minimal AD, residues 737–892) has lost the ability to accumulate in the nucleus under any condition. The region of NirA which is missing in this construct (231–732) also overlaps with the region that was found to be required to block the NES in the *in vitro* co-IP experiments (~550–892).

Taken together, these data led to the model shown in Fig 7B. In the absence of inducer, FmoB (directly or indirectly, indicated by a dotted line) would oxidize Met169 in the NES, this step leading to an open NirA conformation exposing the NES and allowing KapK interaction and nuclear export. In the presence of inducer, Met^ox^169 would be rapidly reduced and this step allows the region between the NES and the AD to block the NES. Whether the inducer acts on the blocking domain itself or on the AD itself remains to be determined. As many modification-of-function mutations as well as intragenic suppressor mutations map to the central region we consider it likely that this portion of NirA carries nitrate-sensitive regulatory amino acids. We therefore termed it NiRD for “Nitrate-Regulatory Domain”.

We have so far not been able to detect direct protein-protein interactions by yeast two-hybrid assays and co-IP using different portions of NirA corresponding to the previously defined baits I, II, III and IV [39]. This failure may be due to the absence of Met^ox^169 in the protein expressed during the yeast double hybrid screen. It is known from calmodulin, (CaM) that Met^ox^ formation leads to drastic conformational changes and uncoupling of interacting domains [40,41].

Additionally Met^ox^ at position 169 might influence the differential formation of disulphide bridges. Although no methionine oxidation is known to be involved in the regulation of Yap1, the *S*. *cerevisiae* regulator of the oxidative stress response, nuclear export of the protein is regulated by a specific disulphide switch which buries or exposes the NES of this activator [42–44].

It is remarkable that, so far, all analysed mutations in the NirA LR-NES sequence (L^165^AGK**M**^169^SNLVL^174^) also influence the function of the protein as a transcription factor. In our previous study we have seen that replacements of leucines 172 and 174 by alanines (L172A/L174A) completely abolish the transcriptional activation function and in this study we determined that isoleucine at position 169 (M169I) partially inactivates the protein (Figs 4D and S4B). In contrast, valine at position 167 (G167V, the NirA^c^1 mutant) or alanine at position 169 (M169A, a mutation created during this work) transform the normally nitrate-dependent transcription factor into a permanently active transcriptional regulator. The suppressor mutations of R347S, all mapped in a relatively short stretch of 50 amino acids between residues 300–350 and included changes to more hydrophobic or electronegative residues (see Table 2). These changes suggested that this small region may form part of the nitrate-regulatory domain (NiRD) which, according to our present model (Fig 7) influences the function of the activation domain.

**Table 2:** Amino acid changes and phenotypes of NirA mutants.

Strain	Partial NiRD Sequence	Function
**R347(A301-K315)**	^301^AKRLILDNDELVNSK^315^…**R** ^347^	wild type, inducible
**R347+V312F**	^301^AKRLILDNDEL**F**NSK^315^…**R** ^347^	Constitutively active
**R347S+K302E**	^301^A**E**RLILDNDELVNSK^315^…**S** ^347^	inducible (CS-suppressor)
**R347S+I305L**	^301^AKRL**L**LDNDELVNSK^315^…**S** ^347^	inducible (CS-suppressor)
**R347S+L306F**	^301^AKRLI**F**DNDELVNSK^315^…**S** ^347^	inducible (CS-suppressor)
**R347S+N308I**	^301^AKRLILD**I**DELVNSK^315^…**S** ^347^	inducible (CS-suppressor)
**R347S+E310A +V312F**	^301^AKRLILDND**A**L**F**NSK^315^…**S** ^347^	inducible (CS-suppressor)
**R347S+K315E**	^301^AKRLILDNDELVNS**E** ^315^…**S** ^347^	inducible (CS-suppressor)
**——————-**	———————————-	———————
**R347(G336-F350)**	^336^GKGWVYSGMSF**R** ^347^MAF^350^	inducible
**R347+K337T**	^336^G**T**GWVYSGMSF**R** ^347^MAF^350^	Constitutively active
**R347S**	^336^GKGWVYSGMSF**S** ^**347**^MAF^350^	non-inducible at 25°C (CS, cryo-sensitive)
**R347S+S342R**	^336^GKGWVY**R**GMSF**S** ^**347**^MAF^350^	inducible (CS-suppressor)
**R347S+M344I**	^336^GKGWVYSG**I**SF**S** ^**347**^MAF^350^	inducible (CS-suppressor)

## Materials and Methods

### *A*. *nidulans* strains, growth conditions and genetic techniques

*A*. *nidulans* strains were grown in *Aspergillus* glucose minimal medium (GMM) and manipulated as previously described [11,17,21]. S1 Table lists all fungal strains used in this study. Some strains are not discussed in the text but were used for sexual crosses to obtain the strains of interest. S2 Table indicates the nucleotide changes in the mutant strains. S3 Table shows all oligonucleotide primers used in this work. Construction of plasmids for gene expression in *A*. *nidulans* and gene knock out methods are described in S1 Text. The strain displaying cold-sensitive (*cs*) growth on nitrate was selected as being unable to grow at 25°C (restrictive temperature) but grows like wild type at 37°C (permissive temperature). Revertants of the *cs* strain were obtained after UV mutagenesis as being able to grow on nitrite and nitrate as sole N-source at 25°C. Strains were backcrossed to a wild type strain (*pabaA*1) to test for intragenic or extragenic reversion events. All revertants showed intragenic suppressor mutations (100% of progeny showed growth on nitrate at the restrictive temperature) and mutations were identified by sequencing of the coding region of *nirA*.

### *E*. *coli* expression, purification and activity determination of recombinant FmoB

cDNA for cloning was prepared as described in S1 Text. FmoB activity was analyzed either by monitoring the NADPH consumption at 340 nm when incubated with different substrates (methionine, cysteine, glutathione, methimazole, N-octylamine), or following product formation, methionine sulfoxide, by HPLC analysis.

### Transcriptional analysis in *A*. *nidulans*

Analysis of nitrate reductase (*niaD*) gene expression in different mutant backgrounds was carried out as described previously [21]. Growth conditions were as follows: strains were grown in AMM containing 0.2% fructose (to de-repress the *alcA* promoter in case of FLAG-*nirA* strains) and 3 mM arginine as a sole nitrogen source for 16 h at 37°C. Mycelia were harvested, washed with AMM and aliquots of the culture were incubated for 30 minutes in nitrogen free (-N) AMM. Subsequently, individual aliquots received either 3 mM arginine (NI) or 10 mM sodium nitrate (IND) and, in case of n-octylamine treatment, additional 2 mM n-octylamine (INDo) (Sigma Aldrich). These cultures were then further incubated on an orbital shaker at 37°C for 20 minutes.

### Fluorescence microscopy

Strains were grown for 16 h at 25°C on cover slips in glucose minimal medium (GMM) and 3 mM arginine as a sole nitrogen source. Compounds were added to the media in the following concentrations: 10 mM n-octylamine, 10 ng/ml leptomycin B (LMB). Germlings were observed by an Olympus Fluoview FV1000 confocal microscope (DAPI: excitation at 405 nm wavelength, emission between 426–479 nm wavelengths; GFP: excitation at 395 nm wavelength, emission at 509 nm wavelength). Microscopy was performed as described [17]. Image manipulation was performed by Image J Fiji software.

### Preparation of cell-free extracts from *A*. *nidulans*

Strains expressing the FLAG-tagged *nirA* gene from the *alcA* promoter were grown on AMM containing 0.2% fructose as carbon source when NirA should be expressed at wild-type levels (TCA extracts). For overexpression of NirA cultures were treated with 50 mM butanone (EMK) for 3 h. For nitrate-induced conditions, 10 mM NaNO_3_ was added and the cells incubated for further 5 minutes. Cells were harvested and cell-free extracts prepared as previously described [45]. For the preparation of whole cell extracts under denaturing conditions (TCA extracts) cells were washed in 20% trichloroacetic acid (TCA), harvested and frozen in liquid nitrogen. Pulverized mycelia was resuspended in 12.5% TCA followed by 3 washing steps with pure acetone. Samples were pelleted, air dried and resuspended in 1x Laemmli Buffer. 15 μg of extracts were loaded per lane. Intensity of signals were calculated with ImageQuant software (Molecular Dynamics) against actin (ActA) (Ab 691001, MP Biomedicals) as loading control. Western blot analysis GFP- and FLAG-tagged of nirA strains, were carried out by preparing total cell extracts according to the methods described by [46]. Grained mycelia were resuspended in protein extraction buffer (20 mM Tris-HCl, pH8, 150 mM NaCl, 0,01% Triton X-100) containing protease inhibitor cocktail (P8340, Sigma). 30 μg per lane of total protein extract was loaded on a 10% acrylamide/bis-acrylamide gel in the presence of β-mercaptoethanol and SDS as denaturing agents. GFP-NirA membranes were incubated with anti-GFP primary Ab (Roche, 11814460001) and with Anti-Mouse IgG (H+L) secondary Ab (Jackson, 115-001-003). FLAG-NirA membranes were incubated with anti-FLAG-tag primary Ab (F1804; Sigma Aldrich) and detected with goat anti-mouse HRP-conjugated secondary Ab (Jackson, 115-035-008).

### Preparation of DNA affinity columns and purification of NirA protein

1 ml Streptavidin Sepharose High Performance (GE Healthcare) slurry was prepared following the manufacturer´s protocol. 30 μg of a biotinylated synthetic 104 bp DNA fragment containing NirA binding site 2 (NirA BS2) [47] in blocking buffer were added and incubated on a rotator over night at 4°C. For NirA protein purification cell extracts of relevant strains were prepared as described above setting the DTT concentration to 0.5 mM in the protein extraction buffers and adding a supplemental centrifugation step at 100,000g for 1 h at 4°C prior to loading the extracts onto the affinity column. Non-specific bound proteins were removed by washing the column with 5 ml washing buffer (25 mM HEPES pH 7.5, 100 mM NaCl, 0.1 mM EDTA, 50 μM ZnCl_2_, 10% glycerol, 0.5 mM DTT). Subsequently NirA BS2 interacting proteins were eluted with 3 ml elution buffer (25 mM HEPES pH 7.5, 50 mM KCl, 5 mM MgCl_2_, 0.1 mM EDTA, 0.5 mM DTT, 10% glycerol, 1 M NaCl, 0.5 mM DTT).

### Mass spectrometric analysis

In-gel protein reduction, alkylation and trypsination were performed as described previously [48]. NanoHPLC-MSMS protein sequencing was performed on the UltiMate system from Dionex. Eluting peptides were directly introduced into the MS and ionized via Pico Tip (New Objective, Cambridge, USA) to be further transferred online to a heated capillary of an ion trap mass spectrometer (LCQ Deca XPplus, Thermo Finnigan). Analysis of MS/MS spectra with respect to peptide identity was routinely performed by applying both the MASCOT (Matrix Science) [49] and the SEQUEST [50] (Bioworks3.1) search engines using the MSDB (MSDB 09082006) and the Global Proteome Machine database (*Aspergillus nidulans*), respectively. The following settings were used for the generation of DTA files and for database search: MW range, 450 – 3500 Da; threshold, 10000; minimum ion count, 10; precursor mass tolerance, ± 3 Da; fragment mass tolerance, ± 0.8 Da; missed cleavages, up to 3; enzyme, trypsin. In general a peptide was reliably identified only if the individual peptide scores were ≥35 (MASCOT) and ≥2 for singly charged, ≥2.5 for doubly charged and ≥3.5 for triply charged peptides (*X*Corr, SEQUEST). The oxidation state of methionine in the NES peptide (methionine or methionine sulfoxide) was regarded as identified, if the particular peptide was identified with both search engines from at least three different experiments.

### *In vitro* peptide interaction studies

Different forms of synthetic biotinylated peptides containing 18 amino acids (DQFESELAGKMSNLVLDG) of the NirA nuclear export sequence (M169 underlined) were synthesized (Biomatik, USA). 1 μmol of the biotinylated peptides were bound to Streptavidin Sepharose Resin (SSR, GA Healthcare) which was equilibrated before by washing it 3 times with binding buffer (20 mM NaH_2_PO_4_, pH 7.4, 100 mM NaCl). After 2 h of incubation with the biotinylated peptides, the supernatant was removed after centrifugation and the SSR washed three times with binding buffer and then equilibrated with protein extraction buffer. The SRR-bound peptides were then incubated with 1 ml of the cell-free extract of the strain Stag-KapK for 2 h on ice. After this incubation period the SRR-peptide-protein complexes were washed twice each with buffer A (150 mM NaCl, 10 mM Tris-HCl, pH 8), buffer B (250 mM NaCl, 10 mM Tris-HCl, pH 8) and buffer C (500 mM NaCl, 10 mM Tris-HCl, pH 8). Samples were denatured in 1x Laemmli buffer, separated on 1.5 mm 10% SDS-PAGE gels and transferred to nitrocellulose membranes for Western blotting. To probe for the presence of S-tag-KapK the membranes were incubated with anti-S-tag primary antibody (Abcam ab19321) and detected with HRP-conjugated rabbit anti-goat secondary Ab (SIGMA A5420).

### Co-immunoprecipitation (Co-IP)

5 μl of the “capture” anti-S-tag antibody (Ab) was first bound to 40 μl protein G agarose beads (PGAB, New England Biolabs) in TSA buffer (10 mM Tris-HCl, pH 8.0; 150 mM NaCl) in a final volume of 800 μl. The mixture was incubated on a spinning wheel at 4°C for 2 h. During this time, the cell-free extracts were pre-cleared to avoid unspecific binding to PGAB. Cell-free extracts of relevant strains were prepared as described above with the modification that the extraction buffer either contained or did not contain 5 mM DTT, depending on thiol-reducing (+DTT) or thiol-non reducing (-DTT) conditions, respectively. For pre-clearing, 1 ml of the resulting protein extracts (5 mg/ml) from strains expressing FLAG-tagged NirA and S-tagged KapK were incubated in the same volume of dilution buffer (100 mM Tris-HCl, pH 8; 5 mM NaCl; 0.1% Triton X-100) containing 50 μl of Protein G Agarose Beads (PGAB, New England Biolabs) for 2 h on ice. The pre-cleared lysates and the linked S-tag- antibody-PGAB complexes were combined after centrifugation and incubated 2 h on ice. After incubation the samples were treated as described above for the immunoprecipitation assays. To detect FLAG-NirA the membranes were incubated with anti-FLAG-tag primary Ab (F1804; Sigma Aldrich) and detected with goat anti-mouse HRP-conjugated secondary Ab (Jackson, 115-035-008). S-tagged KapK was detected as described above. Heavy chain IgG from S-tag primary Ab, used in the Western analysis as loading control of the co-IP samples, was visualized on the same blot during incubation with HRP-conjugated rabbit anti-goat secondary Ab (SIGMA A5420).

## Supporting Information

S1 Fig Methionine sulfoxide reductases MsrA and MsrB and thioredoxin are not required for Met^ox^ reduction and NirA activity. **(A).** Methionine sulfoxide reductases *msrA* and *msrB* deletion mutants show wild-type nuclear localization of NirA. NI, non-induced cells grown on 3 mM arginine as a sole nitrogen source; IND, induced cells treated with 10 mM nitrate. Size bars refer to 5 μm. **(B).** Comparison of *niaD* mRNA levels between the control strain (*alcA*_p_-F*nirA*) and the *msr* double deletion mutant (*alcA*_p_-*FnirA msrAB*ΔΔ). Numbers below the *niaD* hybridization panel are relative values compared to the *niaD* IND expression in the control strain (*alcA*_p_-*FnirA*) that was arbitrarily set to 100. **(C).** NirA is fully functional in thioredoxin mutants. Transcription of the NirA target gene *niiA* was tested in strains carrying a deletion in the single *A*. *nidulans* thioredoxin gene (*trxAΔ*, accession number ANID_00170) under non-induced and induced conditions and compared to wild type (WT), *nirA* ^*c*^1 and a strain in which the *trxA* deletion has been complemented by a functional ectopic copy (27). Growth conditions were as in **B**, except that induction by nitrate was proceeding for 2 hours instead of 20 minutes.
(TIF)

S2 Fig Partial amino acid sequence alignment of several members of the eukaryotic flavin-containing monooxygenases. *A*. *nidulans* FMOs predicted in the *A*. *nidulans* genome database at Broad Institute, www.broadinstitute.org) were selected according to their similarities to human FMO3. FmoB (ANID_04110) showed highest similarity and was subsequently used as query in the local BLAST search. Six additional FMO-type proteins appeared and three of them had predicted domain structures and lengths similar to FmoB. Similarity scores were 2.8e^-39^ for ANID_08206 (designated FmoA), 6.9e^-38^ for ANID_03043 (designated FmoD) and 1.4e^-27^ for ANID_02197 (designated FmoC). Conserved FMO motifs are shown by horizontal black bars, the first conserved GxGxxG motif (Rossman fold, x = any amino acid) for FAD binding, is followed by the FMO-identifying signature FxGxxxHxxxY/F and finally by the less conserved NADP-binding motif GxGxxG (Rossman fold). Large black dots indicate residues that are part of the active site in *Methylophaga* FMO (MeFMO), whereas the black squares mark amino acids that are mutated in patients affected by trimethylaminuria (TMAU) in human FMO3 [51].
(TIF)

S3 Fig Comparison of *nirA* expression levels from native or engineered (*alcA*) promoters. *nirA* mRNA levels were compared between strains expressing the gene from its native promoter or from the *alcA* promoter under carbon de-repressing conditions (0,2% fructose as sole carbon source) and in the presence of 3 mM arginine as sole nitrogen source (except lane IND NO_3_ which contained 10 mM nitrate in addition to arginine). Northern blots show that *nirA* mRNA can barely be detected in Northern hybridizations when expressed from its own, very weak promoter (WT, *nirA* ^c^1) but is clearly seen in strains expressing *nirA* from the de-repressed *alcA* promoter. The mRNA level of the wild type *nirA* gene was tested in the wild type background (lane *alcA*_p_ *-FnirA)* and in the *fmoB* or *msrAmsrB* deletion backgrounds (lanes *alcA*_p_ *-FnirA fmoBΔ* and *alcA*_p_ *-FnirA msrABΔΔ*), respectively. NirA variants carrying replacements of the conserved methionine 169 by alanine (two independent strains were tested in lanes *alcA*_*p*_-*FnirA* ^M169A#1^ and *alcA*_*p*_-*FnirA* ^M169A#2^) or isoleucine (lane *alcA*_*p*_-*FnirA* ^M169I^) were also examined. In all cases in which *nirA* was expressed from the *alcA* promoter, transcripts were clearly detectable and signals similarly strong. Northern hybridization with a probe from the actin (*actA*) gene served as loading control.
(TIF)

S4 Fig Nitrate-independent activity of different NirA variants in the wild type and in the *fmoB*Δ background. We tested the activity of NirA in different variants and genetic backgrounds. **A.** Northern blot shows the *niaD* mRNA level of the nitrate-induced control strain (*alcA*_*p*_ *-FnirA*, IND) which was arbitrarily set to 100% and all other expression levels are relative to this control. These other strains were grown under non-inducing conditions for 16 hours on AMM containing 0.2% fructose (de-repressed conditions for the *alcA* promoter) and 3 mM arginine as a sole nitrogen source. First, we determined if expression of the FLAG-tagged NirA from the derepressed *alcA* promoter renders the wild type protein constitutively active (compare panels WT and *alcA*_p_ *-FnirA*). Whereas the wild type protein is not active without nitrate when expressed from *alcA*_*p*_, the already partially constitutive NirA^c^1 protein is twice as active when expressed from the *alcA* promoter (compare lanes *nirA* ^c^1 and *alcAp*-*FnirA* ^c^1). When expressed from the *alcA* promoter, NirA carrying the M169A exchange (lane *alcA*_*p*_-*FnirA* ^M169A^) is also partially active in the absence of inducer. In contrast, the M169I replacement or deletion of *fmoB* does not lead to a constitutively active FLAG-NirA protein (last two lanes). **B.** Analysis of the *nirA* ^M169I^ variant under induced conditions and after NOC treatment. Northern blot shows the *niaD* mRNA level of the non-induced (NI) and nitrate-induced (IND) control strain (*alcA*_*p*_ *-FnirA*) in which the signal obtained under IND conditions was arbitrarily set to 100%. The NirAM169I variant protein expressed under identical conditions showed only 50% activity as activator of *niaD* expression. As in panel A, the strains were grown under non-inducing conditions for 16 hours on AMM containing 0.2% fructose (de-repressed conditions for the *alcA* promoter) and 3 mM arginine as a sole nitrogen source for non-induced conditions and for induction (IND), 10 mM nitrate was added 2o minutes before harvesting the cells. When the effect of NOC was tested, the compound was added also 20 minutes before harvesting the non-induced cells (NIo) or simultaneously with nitrate in the induced cells (INDo). NOC treatment resulted in a slight reduction of *niaD* mRNA levels of around 25% of the IND level in both strains. In all densitometric measurements, intensity of signals was calculated with ImageQuant and *niaD* expression was normalized with actin (*actA*) expression. Numbers below the *niaD* hybridization panel are the relative values in comparison to the control.
(TIF)

S5 Fig Western blot analysis of NirA variants and in different mutant backgrounds. Evaluation of NirA-GFP and FLAG-NirA protein levels in strains expressing the *nirA*-*gfp* or the *FLAG*-*nirA* constructs from different promoters in the absence (NI) or the presence (IND) of nitrate. 30μg total protein were loaded per lane in the SDS PAGE (for details see Materials and Methods in the main text) **(A).** Western blot analysis shows that the NirA-GFP protein is detected at an apparent molecular size of approximately 130 kDa by the anti-GFP monoclonal antibody (Roche). The fusion protein is highly abundant in all strains expressing the construct from the strong constitutive *gpdA* promoter independently from the presence of nitrate (NI or IND conditions) or the allele of *kapK* present in the strain (*kapK* ^+^ or *kapK*1). Also, the deletion of *fmoA* or *fmoB* did not greatly influence fusion protein abundance (strains *gpdA*_*p*_ *-nirA-gfp fmoA*Δ or *fmoB*Δ), however, *msrA* and *msrB* deletions seemed to have a slightly negative effect on protein amounts (lane *gpdA*_*p*_ *-nirA-gfp msrAB*ΔΔ). Clearly, expression of the fusion construct from the hybrid *ERE-nirA*_*p*_ promoter (EREp under estrogenic conditions, see Materials and Methods) reduced the amount of NirA-GFP under both nitrate induced and non-induced conditions. In comparison to the respective wild type NirA-GFP control, neither NirA-GFP mutants carrying M169A (lane *gpdA*_*p*_-*nirA* ^M169I^–*gfp kapK1*) nor M169I exchanges (lane *ERE*_*p*_-*nirA* ^M169I^–*gfp*) showed lower protein levels. The *nirA637* strain which was used as transformation recipient is used here as negative control for the Western analysis In some protein preparations partial degradation of NirA has occurred which becomes evident as higher mobility band below the main NirA signal in the GFP-Western shown in panel A. **(B).** Western blot analysis after SDS-PAGE of the FLAG-NirA fusion constructs. The FLAG-NirA protein is detected at an apparent molecular size of approximately 105 kDa by the anti-FLAG monoclonal antibody (SIGMA). The fusion protein is expressed from the de-repressed *alcA* promoter (0,2% fructose) in the absence of nitrate (3 mM arginine) and its abundance is similar in all tested strains. Although the same total protein amounts are loaded in panels A and B for SDS PAGE, the lower signal intensity in panel B may not correlated to reduced FLAG-NirA levels because the anti-FLAG antibody may have a lower affinity compared to the GFP Ab. However, the relative strength of the *gpdA* promoter is known to be higher than the de-repressed *alcA* promoter and hence the stronger signal in the GFP Western (panel A) may reflect also higher protein levels.
(TIF)

S6 Fig MS/MS spectra of NirA NES-containing peptides. MS/MS spectra of NirA-NES peptides were derived from FLAG-NirA purified from cells grown under inducing (IND, nitrate 10 mM) or non-inducing (NI, arginine 3 mM) conditions. In each case, analysis was carried out from FLAG-NirA purified from three independently grown cultures. Some mutant strains were grown only under NI conditions to test for the M169 oxidation status of purified FLAG-NirA. All experiments were done in triplicate, one representative graph is shown. Strains and conditions: *1*. *alcA*_p_-*FnirA*: FLAG-NirA expressed in the wild type background and purified from nitrate-induced cultures (IND, 1.1) or non-induced cultures (NI, 1.2). *2*. *alcA*_p_-*FnirA fmoB*Δ: FLAG-NirA expressed in the *fmoB*Δ mutant background and purified from these cells grown under nitrate-induced (IND 2.1-) or non-induced (NI, 2.2) conditions. *3*. *alcA*_p_-*FnirA* ^c^1: FLAG-NirA^c^1 (G167V mutation in the NES) expressed in the wild type background and purified from cells grown under nitrate induced (IND 3.1) or non-induced conditions (NI 3.2).
(DOCX)

S1 Table Strains used in this study. Table shows genotypes, designations and sources of strains used in this study.
(DOCX)

S2 Table Nucleotide changes in the mutant strains. Table shows changes in nirA nucleotide sequences in strains isolated as suppressors of the cold-sensitive *nirA* ^-^ strains. Numbers in the row “position” refer to amino acid positions within the altered proteins, mutations describe the amino acid changes caused by the nucleotide change given in the same lane and number of strains describes how many independent mutants were isolated carrying the described nucleotide change.
(DOCX)

S3 Table Oligonucleotides used in this study. Table shows names and sequences of oligonucleotides used for cloning and biochemical experiments throughout this study.
(DOCX)

S1 Text This section contains supporting experimental procedures and references. (DOCX)

S1 Video Time course of induction of NirA-GFP nuclear localisation. Strain *gpdA*_*p*_-*nirA*-GFP was grown directly on microscope coverslips for 6 h on a non-inducing nitrogen source (3 mM arginine) and subsequently exposed to 10 mM nitrate. Pictures were taken in the laser scanning microscope (scale bar = 10 μm) immediately before addition of nitrate (0 sec) and 20, 40, 60, 80 and 100 seconds after induction.
(AVI)
