# Supplementary figures and images for: Reversible Oxidation of a Conserved Methionine in the Nuclear Export Sequence Determines Subcellular Distribution and Activity of the Fungal Nitrate Regulator NirA
Source: PLoS Genet. 2015 Jul 1;11(7):e1005297. doi: 10.1371/journal.pgen.1005297 (PMC4488483; doi:10.1371/journal.pgen.1005297)

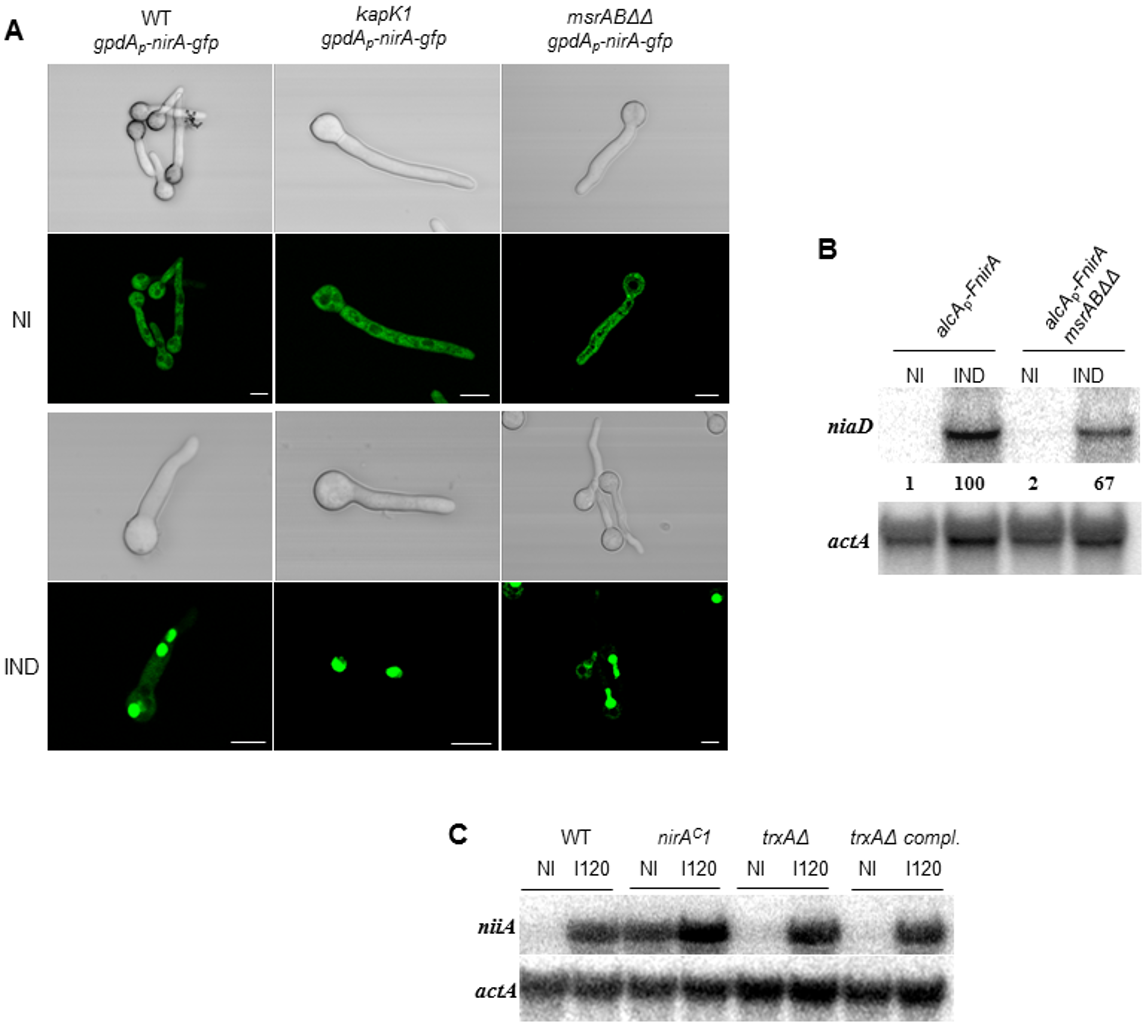

Supplement: S1 Fig — (A). Methionine sulfoxide reductases msrA and msrB deletion mutants show wild-type nuclear localization of NirA. NI, non-induced cells grown on 3 mM arginine as a sole nitrogen source; IND, induced cells treated with 10 mM nitrate. Size bars refer to 5 μm. (B). Comparison of niaD mRNA levels between the control strain (alcA p-FnirA) and the msr double deletion mutant (alcA p-FnirA msrABΔΔ). Numbers below the niaD hybridization panel are relative values compared to the niaD IND expression in the control strain (alcA p-FnirA) that was arbitrarily set to 100. (C). NirA is fully functional in thioredoxin mutants. Transcription of the NirA target gene niiA was tested in strains carrying a deletion in the single A. nidulans thioredoxin gene (trxAΔ, accession number ANID_00170) under non-induced and induced conditions and compared to wild type (WT), nirA c1 and a strain in which the trxA deletion has been complemented by a functional ectopic copy (27). Growth conditions were as in B, except that induction by nitrate was proceeding for 2 hours instead of 20 minutes. (TIF) [file pgen.1005297.s001.tif]

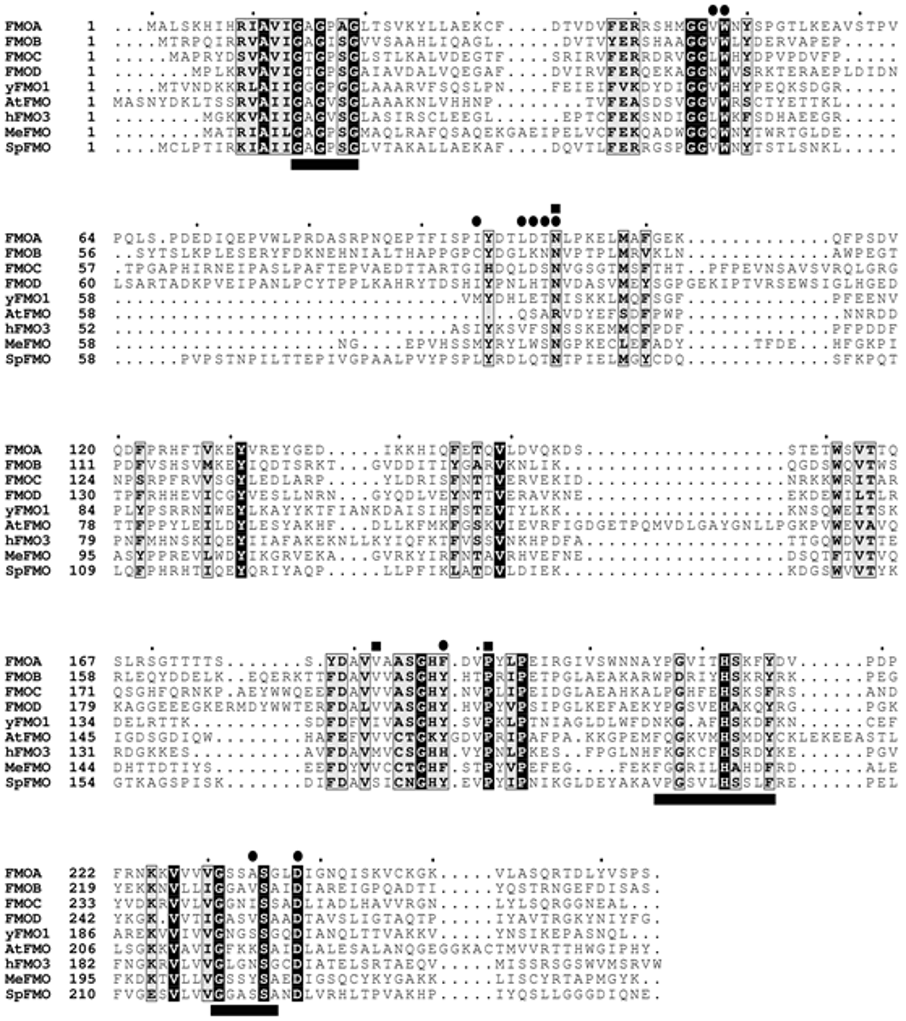

Supplement: S2 Fig — A. nidulans FMOs predicted in the A. nidulans genome database at Broad Institute, www.broadinstitute.org) were selected according to their similarities to human FMO3. FmoB (ANID_04110) showed highest similarity and was subsequently used as query in the local BLAST search. Six additional FMO-type proteins appeared and three of them had predicted domain structures and lengths similar to FmoB. Similarity scores were 2.8e-39 for ANID_08206 (designated FmoA), 6.9e-38 for ANID_03043 (designated FmoD) and 1.4e-27 for ANID_02197 (designated FmoC). Conserved FMO motifs are shown by horizontal black bars, the first conserved GxGxxG motif (Rossman fold, x = any amino acid) for FAD binding, is followed by the FMO-identifying signature FxGxxxHxxxY/F and finally by the less conserved NADP-binding motif GxGxxG (Rossman fold). Large black dots indicate residues that are part of the active site in Methylophaga FMO (MeFMO), whereas the black squares mark amino acids that are mutated in patients affected by trimethylaminuria (TMAU) in human FMO3 [51]. (TIF) [file pgen.1005297.s002.tif]

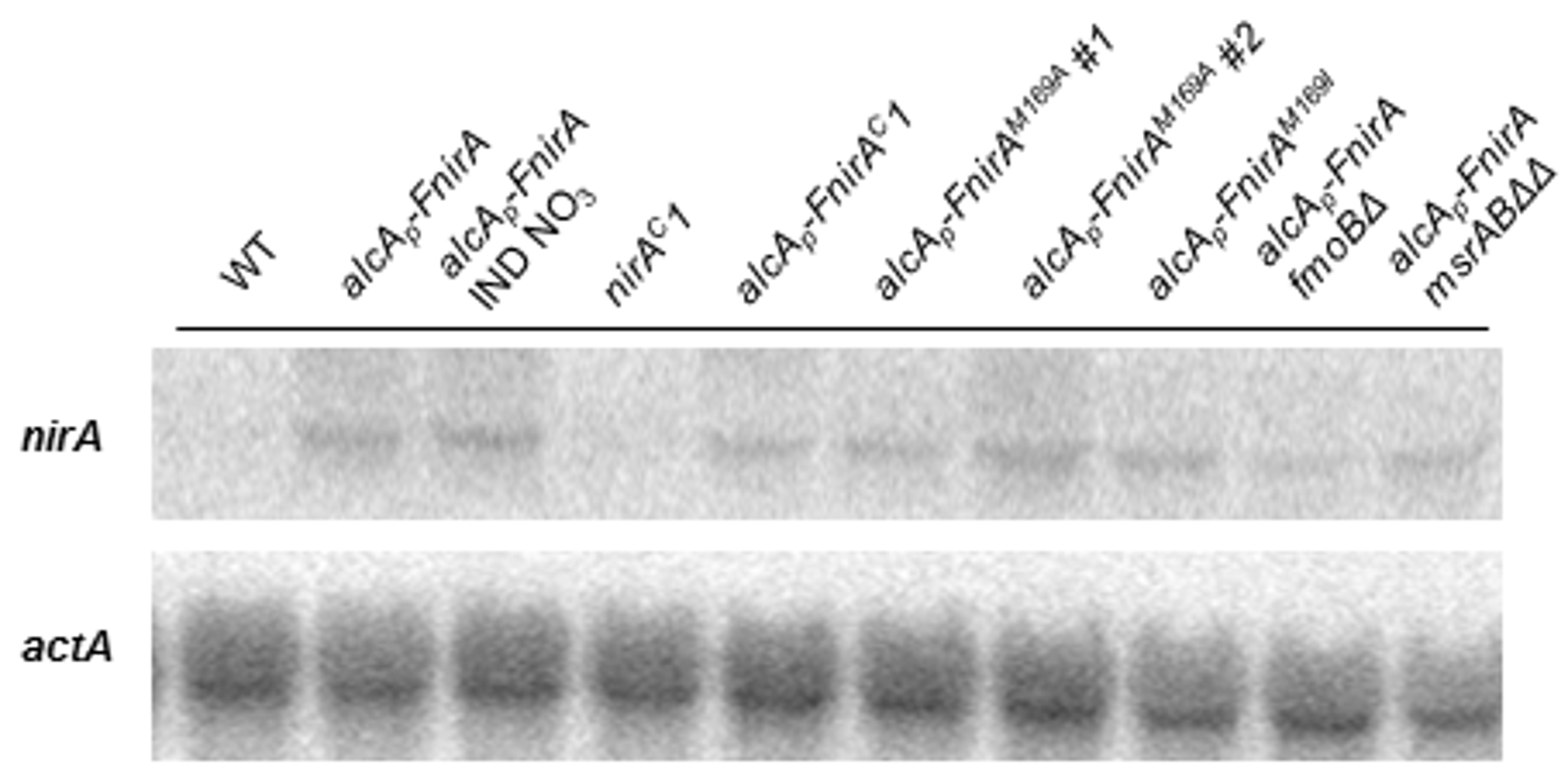

Supplement: S3 Fig — nirA mRNA levels were compared between strains expressing the gene from its native promoter or from the alcA promoter under carbon de-repressing conditions (0,2% fructose as sole carbon source) and in the presence of 3 mM arginine as sole nitrogen source (except lane IND NO3 which contained 10 mM nitrate in addition to arginine). Northern blots show that nirA mRNA can barely be detected in Northern hybridizations when expressed from its own, very weak promoter (WT, nirA c1) but is clearly seen in strains expressing nirA from the de-repressed alcA promoter. The mRNA level of the wild type nirA gene was tested in the wild type background (lane alcA p -FnirA) and in the fmoB or msrAmsrB deletion backgrounds (lanes alcA p -FnirA fmoBΔ and alcA p -FnirA msrABΔΔ), respectively. NirA variants carrying replacements of the conserved methionine 169 by alanine (two independent strains were tested in lanes alcA p-FnirA M169A#1 and alcA p-FnirA M169A#2) or isoleucine (lane alcA p-FnirA M169I) were also examined. In all cases in which nirA was expressed from the alcA promoter, transcripts were clearly detectable and signals similarly strong. Northern hybridization with a probe from the actin (actA) gene served as loading control. (TIF) [file pgen.1005297.s003.tif]

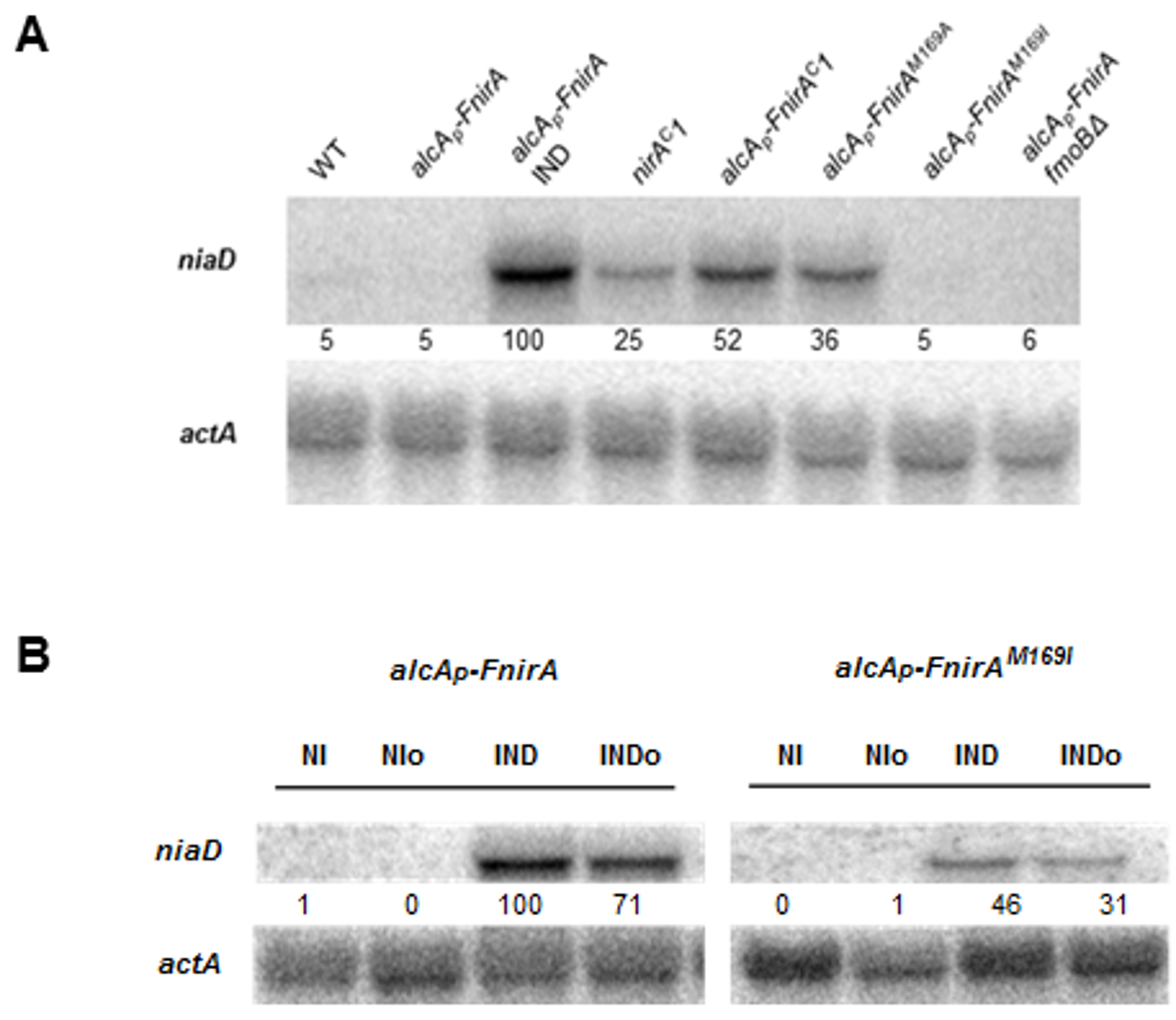

Supplement: S4 Fig — We tested the activity of NirA in different variants and genetic backgrounds. A. Northern blot shows the niaD mRNA level of the nitrate-induced control strain (alcA p -FnirA, IND) which was arbitrarily set to 100% and all other expression levels are relative to this control. These other strains were grown under non-inducing conditions for 16 hours on AMM containing 0.2% fructose (de-repressed conditions for the alcA promoter) and 3 mM arginine as a sole nitrogen source. First, we determined if expression of the FLAG-tagged NirA from the derepressed alcA promoter renders the wild type protein constitutively active (compare panels WT and alcA p -FnirA). Whereas the wild type protein is not active without nitrate when expressed from alcA p, the already partially constitutive NirAc1 protein is twice as active when expressed from the alcA promoter (compare lanes nirA c1 and alcAp-FnirA c1). When expressed from the alcA promoter, NirA carrying the M169A exchange (lane alcA p-FnirA M169A) is also partially active in the absence of inducer. In contrast, the M169I replacement or deletion of fmoB does not lead to a constitutively active FLAG-NirA protein (last two lanes). B. Analysis of the nirA M169I variant under induced conditions and after NOC treatment. Northern blot shows the niaD mRNA level of the non-induced (NI) and nitrate-induced (IND) control strain (alcA p -FnirA) in which the signal obtained under IND conditions was arbitrarily set to 100%. The NirAM169I variant protein expressed under identical conditions showed only 50% activity as activator of niaD expression. As in panel A, the strains were grown under non-inducing conditions for 16 hours on AMM containing 0.2% fructose (de-repressed conditions for the alcA promoter) and 3 mM arginine as a sole nitrogen source for non-induced conditions and for induction (IND), 10 mM nitrate was added 2o minutes before harvesting the cells. When the effect of NOC was tested, the compound was added also 20 minutes before harv [file pgen.1005297.s004.tif]

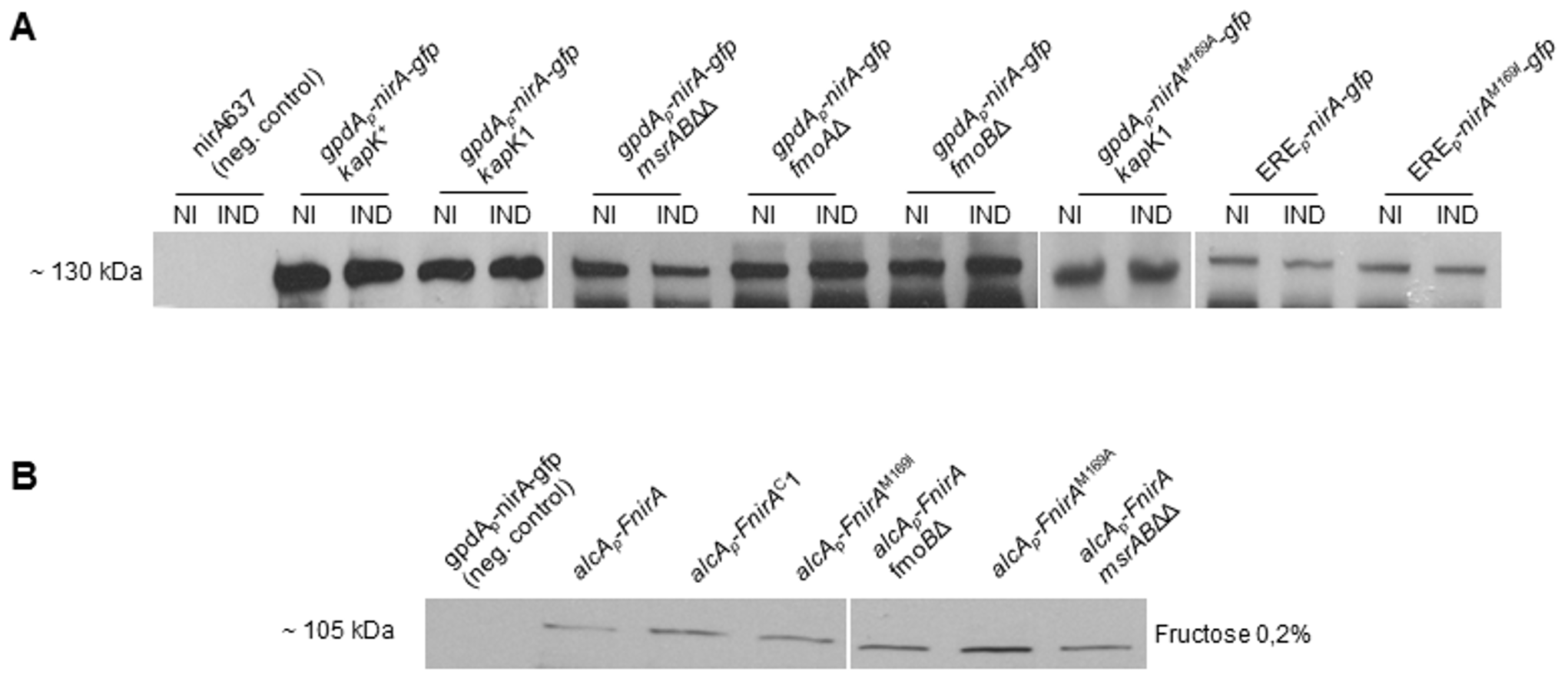

Supplement: S5 Fig — Evaluation of NirA-GFP and FLAG-NirA protein levels in strains expressing the nirA-gfp or the FLAG-nirA constructs from different promoters in the absence (NI) or the presence (IND) of nitrate. 30μg total protein were loaded per lane in the SDS PAGE (for details see Materials and Methods in the main text) (A). Western blot analysis shows that the NirA-GFP protein is detected at an apparent molecular size of approximately 130 kDa by the anti-GFP monoclonal antibody (Roche). The fusion protein is highly abundant in all strains expressing the construct from the strong constitutive gpdA promoter independently from the presence of nitrate (NI or IND conditions) or the allele of kapK present in the strain (kapK + or kapK1). Also, the deletion of fmoA or fmoB did not greatly influence fusion protein abundance (strains gpdA p -nirA-gfp fmoAΔ or fmoBΔ), however, msrA and msrB deletions seemed to have a slightly negative effect on protein amounts (lane gpdA p -nirA-gfp msrABΔΔ). Clearly, expression of the fusion construct from the hybrid ERE-nirA p promoter (EREp under estrogenic conditions, see Materials and Methods) reduced the amount of NirA-GFP under both nitrate induced and non-induced conditions. In comparison to the respective wild type NirA-GFP control, neither NirA-GFP mutants carrying M169A (lane gpdA p-nirA M169I–gfp kapK1) nor M169I exchanges (lane ERE p-nirA M169I–gfp) showed lower protein levels. The nirA637 strain which was used as transformation recipient is used here as negative control for the Western analysis In some protein preparations partial degradation of NirA has occurred which becomes evident as higher mobility band below the main NirA signal in the GFP-Western shown in panel A. (B). Western blot analysis after SDS-PAGE of the FLAG-NirA fusion constructs. The FLAG-NirA protein is detected at an apparent molecular size of approximately 105 kDa by the anti-FLAG monoclonal antibody (SIGMA). The fusion protein is expressed from the de-repressed alcA prom [file pgen.1005297.s005.tif]
