# Supplementary material for: Reversible Oxidation of a Conserved Methionine in the Nuclear Export Sequence Determines Subcellular Distribution and Activity of the Fungal Nitrate Regulator NirA
Source: PLoS Genet. 2015 Jul 1;11(7):e1005297. doi: 10.1371/journal.pgen.1005297 (PMC4488483; doi:10.1371/journal.pgen.1005297)
Supplement: S6 Fig — MS/MS spectra of NirA-NES peptides were derived from FLAG-NirA purified from cells grown under inducing (IND, nitrate 10 mM) or non-inducing (NI, arginine 3 mM) conditions. In each case, analysis was carried out from FLAG-NirA purified from three independently grown cultures. Some mutant strains were grown only under NI conditions to test for the M169 oxidation status of purified FLAG-NirA. All experiments were done in triplicate, one representative graph is shown. Strains and conditions: 1. alcA p-FnirA: FLAG-NirA expressed in the wild type background and purified from nitrate-induced cultures (IND, 1.1) or non-induced cultures (NI, 1.2). 2. alcA p-FnirA fmoBΔ: FLAG-NirA expressed in the fmoBΔ mutant background and purified from these cells grown under nitrate-induced (IND 2.1-) or non-induced (NI, 2.2) conditions. 3. alcA p-FnirA c1: FLAG-NirAc1 (G167V mutation in the NES) expressed in the wild type background and purified from cells grown under nitrate induced (IND 3.1) or non-induced conditions (NI 3.2). (DOCX) [file pgen.1005297.s006.docx]

**Figure S6. MS/MS spectra of NirA NES-containing peptides.**

**1. *alcA*_p_-*FnirA***

**1.1 *alcA*_p_-*FnirA* IND**

MS/MS Fragmentation of **MSNLVLDGSR**
Found in **Q5BH82_EMENI**, NIRA_EMENI NITROGEN ASSIMILATION TRANSCRIPTION FACTOR NIRA.- Emericella nidulans (Aspergillus nidulans).

Match to Query 5: 1090.699774 from (546.357163,2+)


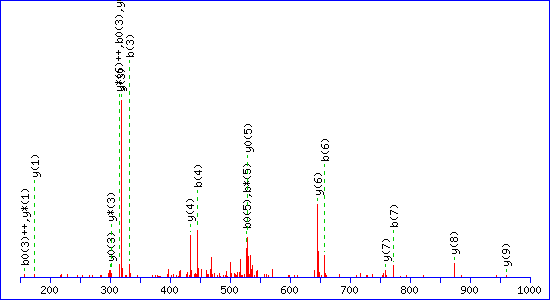


**Monoisotopic mass of neutral peptide Mr(calc):** 1090.5441

**Fixed modifications:** Carbamidomethyl (C)

**Ions Score:** 54 **Expect:** 0.014

**Matches (Bold Red):** 21/100 fragment ions using 35 most intense peaks

| **#** | **b** | **b^++^** | **b*** | **b*^++^** | **b^0^** | **b^0++^** | **Seq.** | **y** | **y^++^** | **y*** | **y*^++^** | **y^0^** | **y^0++^** | **#** |
| --- | --- | --- | --- | --- | --- | --- | --- | --- | --- | --- | --- | --- | --- | --- |
| **1** | 132.0478 | 66.5275 |  |  |  |  | **M** |  |  |  |  |  |  | **10** |
| **2** | 219.0798 | 110.0435 |  |  | 201.0692 | 101.0382 | **S** | **960.5109** | 480.7591 | 943.4843 | 472.2458 | 942.5003 | 471.7538 | **9** |
| **3** | **333.1227** | 167.0650 | 316.0962 | 158.5517 | **315.1122** | **158.0597** | **N** | **873.4789** | 437.2431 | 856.4523 | 428.7298 | 855.4683 | 428.2378 | **8** |
| **4** | **446.2068** | 223.6070 | 429.1802 | 215.0938 | 428.1962 | 214.6017 | **L** | **759.4359** | 380.2216 | 742.4094 | 371.7083 | 741.4254 | 371.2163 | **7** |
| **5** | 545.2752 | 273.1412 | **528.2486** | 264.6280 | **527.2646** | 264.1360 | **V** | **646.3519** | 323.6796 | 629.3253 | **315.1663** | 628.3413 | **314.6743** | **6** |
| **6** | **658.3593** | 329.6833 | 641.3327 | 321.1700 | 640.3487 | 320.6780 | **L** | 547.2835 | 274.1454 | 530.2569 | 265.6321 | **529.2729** | 265.1401 | **5** |
| **7** | **773.3862** | 387.1967 | 756.3597 | 378.6835 | 755.3756 | 378.1915 | **D** | **434.1994** | 217.6033 | 417.1728 | 209.0901 | 416.1888 | 208.5980 | **4** |
| **8** | 830.4077 | 415.7075 | 813.3811 | 407.1942 | 812.3971 | 406.7022 | **G** | **319.1724** | 160.0899 | **302.1459** | 151.5766 | **301.1619** | 151.0846 | **3** |
| **9** | 917.4397 | 459.2235 | 900.4131 | 450.7102 | 899.4291 | 450.2182 | **S** | 262.1510 | 131.5791 | 245.1244 | 123.0659 | 244.1404 | 122.5738 | **2** |
| **10** |  |  |  |  |  |  | **R** | **175.1190** | 88.0631 | **158.0924** | 79.5498 |  |  | **1** |

**1.2 *alcA*_p_-*FnirA* NI**

MS/MS Fragmentation of **MSNLVLDGSR**
Found in **Q5BH82_EMENI**, NIRA_EMENI NITROGEN ASSIMILATION TRANSCRIPTION FACTOR NIRA.- Emericella nidulans (Aspergillus nidulans).

Match to Query 3: 1107.232074 from (554.623313,2+)


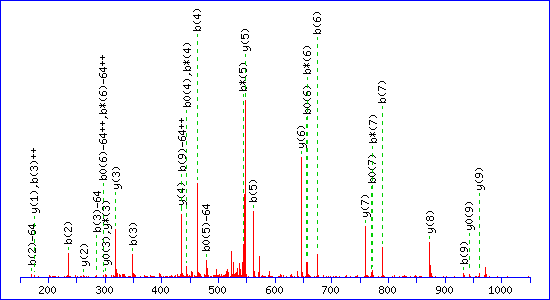


**Monoisotopic mass of neutral peptide Mr(calc):** 1106.5390

**Fixed modifications:** Carbamidomethyl (C)

**Variable modifications:**

**M1:** Oxidation (M), with neutral losses 0.0000(shown in table), 63.9983

**Ions Score:** 49 **Expect:** 0.041

**Matches (Bold Red):** 33/148 fragment ions using 73 most intense peaks

| **#** | **b** | **b^++^** | **b*** | **b*^++^** | **b^0^** | **b^0++^** | **Seq.** | **y** | **y^++^** | **y*** | **y*^++^** | **y^0^** | **y^0++^** | **#** |
| --- | --- | --- | --- | --- | --- | --- | --- | --- | --- | --- | --- | --- | --- | --- |
| **1** | 148.0427 | 74.5250 |  |  |  |  | **M** |  |  |  |  |  |  | **10** |
| **2** | **235.0747** | 118.0410 |  |  | 217.0641 | 109.0357 | **S** | **960.5109** | 480.7591 | 943.4843 | 472.2458 | **942.5003** | 471.7538 | **9** |
| **3** | **349.1176** | **175.0625** | 332.0911 | 166.5492 | 331.1071 | 166.0572 | **N** | **873.4789** | 437.2431 | 856.4523 | 428.7298 | 855.4683 | 428.2378 | **8** |
| **4** | **462.2017** | 231.6045 | **445.1752** | 223.0912 | **444.1911** | 222.5992 | **L** | **759.4359** | 380.2216 | 742.4094 | 371.7083 | 741.4254 | 371.2163 | **7** |
| **5** | **561.2701** | 281.1387 | **544.2436** | 272.6254 | 543.2595 | 272.1334 | **V** | **646.3519** | 323.6796 | 629.3253 | 315.1663 | 628.3413 | 314.6743 | **6** |
| **6** | **674.3542** | 337.6807 | **657.3276** | 329.1675 | **656.3436** | 328.6754 | **L** | **547.2835** | 274.1454 | 530.2569 | 265.6321 | 529.2729 | 265.1401 | **5** |
| **7** | **789.3811** | 395.1942 | **772.3546** | 386.6809 | **771.3706** | 386.1889 | **D** | **434.1994** | 217.6033 | 417.1728 | 209.0901 | 416.1888 | 208.5980 | **4** |
| **8** | 846.4026 | 423.7049 | 829.3760 | 415.1917 | 828.3920 | 414.6996 | **G** | **319.1724** | 160.0899 | **302.1459** | 151.5766 | **301.1619** | 151.0846 | **3** |
| **9** | **933.4346** | 467.2209 | 916.4081 | 458.7077 | 915.4240 | 458.2157 | **S** | **262.1510** | 131.5791 | 245.1244 | 123.0659 | 244.1404 | 122.5738 | **2** |
| **10** |  |  |  |  |  |  | **R** | **175.1190** | 88.0631 | 158.0924 | 79.5498 |  |  | **1** |

**2. *alcA*_p_*-FnirA* *fmoB*Δ**

**2.1 *alcA*_p_-*FnirA fmoB*Δ IND**

MS/MS Fragmentation of **MSNLVLDGSR**
Found in **Q5BH82_EMENI**, NIRA_EMENI NITROGEN ASSIMILATION TRANSCRIPTION FACTOR NIRA.- Emericella nidulans (Aspergillus nidulans).

Match to Query 1: 1091.207674 from (546.611113,2+)


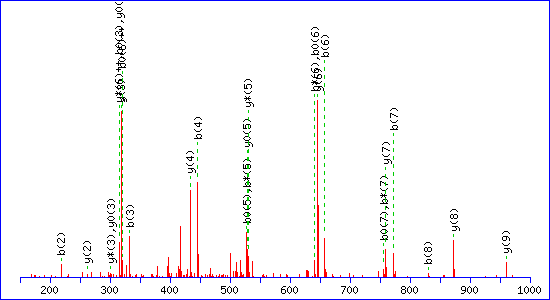


**Monoisotopic mass of neutral peptide Mr(calc):** 1090.5441

**Fixed modifications:** Carbamidomethyl (C)

**Ions Score:** 52 **Expect:** 0.024

**Matches (Bold Red):** 27/100 fragment ions using 40 most intense peaks

| **#** | **b** | **b^++^** | **b*** | **b*^++^** | **b^0^** | **b^0++^** | **Seq.** | **y** | **y^++^** | **y*** | **y*^++^** | **y^0^** | **y^0++^** | **#** |
| --- | --- | --- | --- | --- | --- | --- | --- | --- | --- | --- | --- | --- | --- | --- |
| **1** | 132.0478 | 66.5275 |  |  |  |  | **M** |  |  |  |  |  |  | **10** |
| **2** | **219.0798** | 110.0435 |  |  | 201.0692 | 101.0382 | **S** | **960.5109** | 480.7591 | 943.4843 | 472.2458 | 942.5003 | 471.7538 | **9** |
| **3** | **333.1227** | 167.0650 | 316.0962 | 158.5517 | **315.1122** | 158.0597 | **N** | **873.4789** | 437.2431 | 856.4523 | 428.7298 | 855.4683 | 428.2378 | **8** |
| **4** | **446.2068** | 223.6070 | 429.1802 | 215.0938 | 428.1962 | 214.6017 | **L** | **759.4359** | 380.2216 | 742.4094 | 371.7083 | 741.4254 | 371.2163 | **7** |
| **5** | 545.2752 | 273.1412 | **528.2486** | 264.6280 | **527.2646** | 264.1360 | **V** | **646.3519** | 323.6796 | 629.3253 | **315.1663** | 628.3413 | **314.6743** | **6** |
| **6** | **658.3593** | 329.6833 | **641.3327** | 321.1700 | **640.3487** | **320.6780** | **L** | 547.2835 | 274.1454 | **530.2569** | 265.6321 | **529.2729** | 265.1401 | **5** |
| **7** | **773.3862** | 387.1967 | **756.3597** | 378.6835 | **755.3756** | 378.1915 | **D** | **434.1994** | 217.6033 | 417.1728 | 209.0901 | 416.1888 | 208.5980 | **4** |
| **8** | **830.4077** | 415.7075 | 813.3811 | 407.1942 | 812.3971 | 406.7022 | **G** | **319.1724** | 160.0899 | **302.1459** | 151.5766 | **301.1619** | 151.0846 | **3** |
| **9** | 917.4397 | 459.2235 | 900.4131 | 450.7102 | 899.4291 | 450.2182 | **S** | **262.1510** | 131.5791 | 245.1244 | 123.0659 | 244.1404 | 122.5738 | **2** |
| **10** |  |  |  |  |  |  | **R** | 175.1190 | 88.0631 | 158.0924 | 79.5498 |  |  | **1** |

**2.4 *alcA*_p_-*FnirA fmoB*Δ NI**

MS/MS Fragmentation of **MSNLVLDGSR**
Found in **Q5BH82_EMENI**, NIRA_EMENI NITROGEN ASSIMILATION TRANSCRIPTION FACTOR NIRA.- Emericella nidulans (Aspergillus nidulans).

Match to Query 2: 1091.149574 from (546.582063,2+)


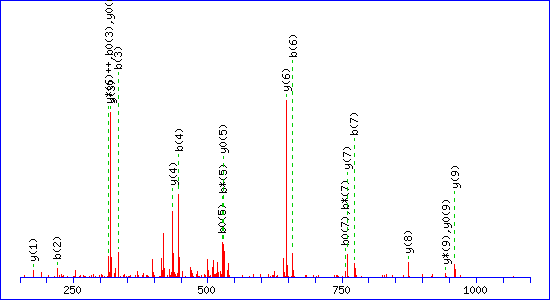


**Monoisotopic mass of neutral peptide Mr(calc):** 1090.5441

**Fixed modifications:** Carbamidomethyl (C)

**Ions Score:** 59 **Expect:** 0.0058

**Matches (Bold Red):** 22/100 fragment ions using 28 most intense peaks

|  | **b** | **b^++^** | **b*** | **b*^++^** | **b^0^** | **b^0++^** | **Seq.** | **y** | **y^++^** | **y*** | **y*^++^** | **y^0^** | **y^0++^** | **#** |
| --- | --- | --- | --- | --- | --- | --- | --- | --- | --- | --- | --- | --- | --- | --- |
| **1** | 132.0478 | 66.5275 |  |  |  |  | **M** |  |  |  |  |  |  | **10** |
| **2** | **219.0798** | 110.0435 |  |  | 201.0692 | 101.0382 | **S** | **960.5109** | 480.7591 | **943.4843** | 472.2458 | **942.5003** | 471.7538 | **9** |
| **3** | **333.1227** | 167.0650 | 316.0962 | 158.5517 | **315.1122** | 158.0597 | **N** | **873.4789** | 437.2431 | 856.4523 | 428.7298 | 855.4683 | 428.2378 | **8** |
| **4** | **446.2068** | 223.6070 | 429.1802 | 215.0938 | 428.1962 | 214.6017 | **L** | **759.4359** | 380.2216 | 742.4094 | 371.7083 | 741.4254 | 371.2163 | **7** |
| **5** | 545.2752 | 273.1412 | **528.2486** | 264.6280 | **527.2646** | 264.1360 | **V** | **646.3519** | 323.6796 | 629.3253 | **315.1663** | 628.3413 | **314.6743** | **6** |
| **6** | **658.3593** | 329.6833 | 641.3327 | 321.1700 | 640.3487 | 320.6780 | **L** | 547.2835 | 274.1454 | 530.2569 | 265.6321 | **529.2729** | 265.1401 | **5** |
| **7** | **773.3862** | 387.1967 | **756.3597** | 378.6835 | **755.3756** | 378.1915 | **D** | **434.1994** | 217.6033 | 417.1728 | 209.0901 | 416.1888 | 208.5980 | **4** |
| **8** | 830.4077 | 415.7075 | 813.3811 | 407.1942 | 812.3971 | 406.7022 | **G** | **319.1724** | 160.0899 | 302.1459 | 151.5766 | 301.1619 | 151.0846 | **3** |
| **9** | 917.4397 | 459.2235 | 900.4131 | 450.7102 | 899.4291 | 450.2182 | **S** | 262.1510 | 131.5791 | 245.1244 | 123.0659 | 244.1404 | 122.5738 | **2** |
| **10** |  |  |  |  |  |  | **R** | **175.1190** | 88.0631 | 158.0924 | 79.5498 |  |  | **1** |

**3. *alcA*_p_- *FnirA*^c^1**

**3.1 *alcA*_p_-*FnirA*^c^1 IND**

MS/MS Fragmentation of **MSNLVLDGSR**
Found in **Q5BH82_EMENI**, NIRA_EMENI NITROGEN ASSIMILATION TRANSCRIPTION FACTOR NIRA.- Emericella nidulans (Aspergillus nidulans).

Match to Query 1: 1091.030874 from (546.522713,2+)


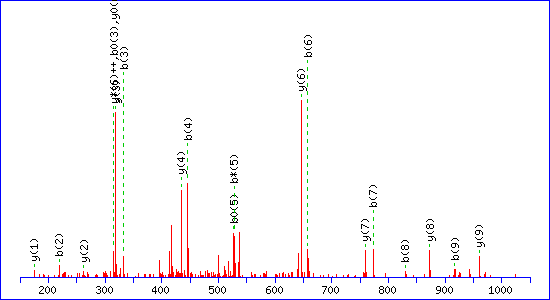


**Monoisotopic mass of neutral peptide Mr(calc):** 1090.5441

**Fixed modifications:** Carbamidomethyl (C)

**Ions Score:** 69 **Expect:** 0.004

**Matches (Bold Red):** 20/100 fragment ions using 31 most intense peaks

| **#** | **b** | **b^++^** | **b*** | **b*^++^** | **b^0^** | **b^0++^** | **Seq.** | **y** | **y^++^** | **y*** | **y*^++^** | **y^0^** | **y^0++^** | **#** |
| --- | --- | --- | --- | --- | --- | --- | --- | --- | --- | --- | --- | --- | --- | --- |
| **1** | 132.0478 | 66.5275 |  |  |  |  | **M** |  |  |  |  |  |  | **10** |
| **2** | **219.0798** | 110.0435 |  |  | 201.0692 | 101.0382 | **S** | **960.5109** | 480.7591 | 943.4843 | 472.2458 | 942.5003 | 471.7538 | **9** |
| **3** | **333.1227** | 167.0650 | 316.0962 | 158.5517 | **315.1122** | 158.0597 | **N** | **873.4789** | 437.2431 | 856.4523 | 428.7298 | 855.4683 | 428.2378 | **8** |
| **4** | **446.2068** | 223.6070 | 429.1802 | 215.0938 | 428.1962 | 214.6017 | **L** | **759.4359** | 380.2216 | 742.4094 | 371.7083 | 741.4254 | 371.2163 | **7** |
| **5** | 545.2752 | 273.1412 | **528.2486** | 264.6280 | **527.2646** | 264.1360 | **V** | **646.3519** | 323.6796 | 629.3253 | **315.1663** | 628.3413 | **314.6743** | **6** |
| **6** | **658.3593** | 329.6833 | 641.3327 | 321.1700 | 640.3487 | 320.6780 | **L** | 547.2835 | 274.1454 | 530.2569 | 265.6321 | 529.2729 | 265.1401 | **5** |
| **7** | **773.3862** | 387.1967 | 756.3597 | 378.6835 | 755.3756 | 378.1915 | **D** | **434.1994** | 217.6033 | 417.1728 | 209.0901 | 416.1888 | 208.5980 | **4** |
| **8** | **830.4077** | 415.7075 | 813.3811 | 407.1942 | 812.3971 | 406.7022 | **G** | **319.1724** | 160.0899 | 302.1459 | 151.5766 | 301.1619 | 151.0846 | **3** |
| **9** | **917.4397** | 459.2235 | 900.4131 | 450.7102 | 899.4291 | 450.2182 | **S** | **262.1510** | 131.5791 | 245.1244 | 123.0659 | 244.1404 | 122.5738 | **2** |
| **10** |  |  |  |  |  |  | **R** | **175.1190** | 88.0631 | 158.0924 | 79.5498 |  |  | **1** |

**3.4 *alcA*_p_-*FnirA*^c^1 NI**

MS/MS Fragmentation of **MSNLVLDGSR**
Found in **Q5BH82_EMENI**, NIRA_EMENI NITROGEN ASSIMILATION TRANSCRIPTION FACTOR NIRA.- Emericella nidulans (Aspergillus nidulans).

Match to Query 1: 1091.741274 from (546.877913,2+)


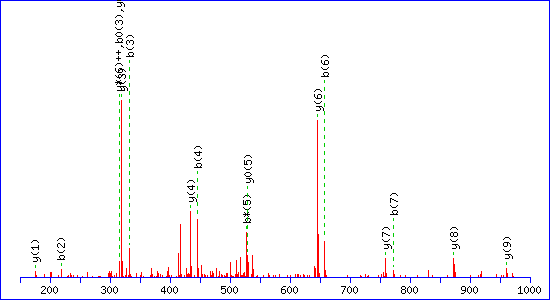


**Monoisotopic mass of neutral peptide Mr(calc):** 1090.5441

**Fixed modifications:** Carbamidomethyl (C)

**Ions Score:** 64 **Expect:** 0.012

**Matches (Bold Red):** 17/100 fragment ions using 19 most intense peaks

| **#** | **b** | **b^++^** | **b*** | **b*^++^** | **b^0^** | **b^0++^** | **Seq.** | **y** | **y^++^** | **y*** | **y*^++^** | **y^0^** | **y^0++^** | **#** |
| --- | --- | --- | --- | --- | --- | --- | --- | --- | --- | --- | --- | --- | --- | --- |
| **1** | 132.0478 | 66.5275 |  |  |  |  | **M** |  |  |  |  |  |  | **10** |
| **2** | **219.0798** | 110.0435 |  |  | 201.0692 | 101.0382 | **S** | **960.5109** | 480.7591 | 943.4843 | 472.2458 | 942.5003 | 471.7538 | **9** |
| **3** | **333.1227** | 167.0650 | 316.0962 | 158.5517 | **315.1122** | 158.0597 | **N** | **873.4789** | 437.2431 | 856.4523 | 428.7298 | 855.4683 | 428.2378 | **8** |
| **4** | **446.2068** | 223.6070 | 429.1802 | 215.0938 | 428.1962 | 214.6017 | **L** | **759.4359** | 380.2216 | 742.4094 | 371.7083 | 741.4254 | 371.2163 | **7** |
| **5** | 545.2752 | 273.1412 | **528.2486** | 264.6280 | 527.2646 | 264.1360 | **V** | **646.3519** | 323.6796 | 629.3253 | **315.1663** | 628.3413 | **314.6743** | **6** |
| **6** | **658.3593** | 329.6833 | 641.3327 | 321.1700 | 640.3487 | 320.6780 | **L** | 547.2835 | 274.1454 | 530.2569 | 265.6321 | **529.2729** | 265.1401 | **5** |
| **7** | **773.3862** | 387.1967 | 756.3597 | 378.6835 | 755.3756 | 378.1915 | **D** | **434.1994** | 217.6033 | 417.1728 | 209.0901 | 416.1888 | 208.5980 | **4** |
| **8** | 830.4077 | 415.7075 | 813.3811 | 407.1942 | 812.3971 | 406.7022 | **G** | **319.1724** | 160.0899 | 302.1459 | 151.5766 | 301.1619 | 151.0846 | **3** |
| **9** | 917.4397 | 459.2235 | 900.4131 | 450.7102 | 899.4291 | 450.2182 | **S** | 262.1510 | 131.5791 | 245.1244 | 123.0659 | 244.1404 | 122.5738 | **2** |
| **10** |  |  |  |  |  |  | **R** | **175.1190** | 88.0631 | 158.0924 | 79.5498 |  |  | **1** |
