# Supplementary material for: Reversible Oxidation of a Conserved Methionine in the Nuclear Export Sequence Determines Subcellular Distribution and Activity of the Fungal Nitrate Regulator NirA
Source: PLoS Genet. 2015 Jul 1;11(7):e1005297. doi: 10.1371/journal.pgen.1005297 (PMC4488483; doi:10.1371/journal.pgen.1005297)
Supplement: S1 Table — Table shows genotypes, designations and sources of strains used in this study. (DOCX) [file pgen.1005297.s007.docx]

**Table S1. Strains used in this study.**

| **Strains** | **Genotype/comment** | **Source** |
| --- | --- | --- |
| Wild type (WT) | *veA*1, *pabaA*1, *nirA*^+^, *kapK*^+^ | FGSC* |
| SAAIII | *veA*1, *wA*3, *argB*2*, biA*1*, pyroA*4*, riboA*1 | FGSC* |
| *gpdA*_p_*-fmoB-gfp* | *veA*1, *wA*3, *argB*2*, biA*1*, pyroA*4*, riboA*1, *gpdA*_p_*-fmoB-gfp::pyroA*3/4 | this work |
| *nirA*637 | *veA*1, *pabaA*1, *nirA*637 (NirA in-phase deletion of 153 amino acids after the first methionine) | (Muro-Pastor et al., 1999) |
| *nirA*637 | *veA*1, *wA*2, *pabaA*1*, pyrG*89, *nirA*637 | this work |
| *nirA*^c^1 | *veA*1, *biA*1, *nirA*^c^1 (G167V) | (Bernreiter et al., 2007) |
| *kapK*1 | *veA*1, *wa*2*, pabaA*1*, pyroA*4*, gpdA*_p_*-nirA-gfp, kapK*1(T525C)*::pyr*4*::kapK*^+^ (T525C, sensitive to leptomycin B) | (Bernreiter et al., 2007) |
| *kapK*1 *fmoA*Δ | *veA*1, *wa*2*, pabaA*1*, pyroA*4*, gpdA*_p_*-nirA*-*gfp*, *kapK*1(T525C)*::pyr*4*::kapK*^+^; *fmoA*Δ*::pyroA* | this work |
| *kapK*1 *fmoB*Δ | *veA*1, *wa*2*, pabaA*1*, pyroA*4*, gpdA*_p_*-nirA*-*gfp*, *kapK*1(T525C)*::pyr*4*::kapK*^+^; *fmoB*Δ*::pyroA* | this work |
| *kapK*1 *nirA*^M169A^ | *veA*1, *wa*2, *pabaA*1*, pyroA*4, *pyrG*89, *nirA*637, *gpdA*_p_*-nirA*(M169A)-*gfp*::*pyroA*3/4, *kapK*(T525C)*::pyr4::kapK* | this work |
| kap*K*1 *nirA*^ADΔ^ | *veA*1, *wa*2, *pabaA*1*, pyroA*4, *pyrG*89, *nirA*637, *gpdA*_p_*-nirA-*ADΔ-*gfp::pyroA*3/4, *kapK*(T525C)*::pyr4::kapK* (lacking amino acids 700-892, corresponding to the activation domain of NirA) | this work |
| ERE_p_-*nirA*-*gfp* | *veA*1, *pyroA*4*, riboA*1 *pyrG*89*, phER-pyr4, ERE-RS-nirA*_p_*-nirA-gfp* | (Bernreiter et al., 2007) |
| ERE_p_-*nirA*^M169I^-*gfp* | *veA*1, *pyroA*4*, riboA*1, *pyrG*89*, phER-pyr4, ERE-RS-nirA*_p_*-nirA*(M169I)*-gfp* | this work |
| *alcA*_p_-*FnirA* | *veA*1, *pabaA*1, *nirA*637:: *alcA*_p_*Flag*-*nirA* | (Berger et al., 2006) |
| *alcA*_p_-*FnirA fmoB*Δ | *veA*1, *pabaA*1, *pantoB*100, *nirA*637::*alcA*_p_*Flag*-*nirA*, *fmoB*Δ | this work |
| *alcA*_p_-*FnirA*^M169I^ | *veA*1, *pabaA*1, *nirA*637::*alcA*_p_*Flag*-*nirA*(M169I) | this work |
| *alcA*_p_-*FnirA*^M169A^ | *veA*1, *pabaA*1, *nirA*637::*alcA*_p_*Flag*-*nirA*(M169A) | this work |
| *alcA*_p_-*FnirA*^c^1 | *veA*1, *pabaA*1, *nirA*637:: *alcA*_p_*Flag*-*nirA*^c^1 | this work |
| *msrAB*ΔΔ | *veA*1, *pyrG*89*; argB*2*, pyroA*4, *msrA*Δ*::pyrG, msrB*Δ*::pyroA* | (Soriani et al., 2009) |
| *gpdA*_p_*-nirA*-*gfp msrAB*ΔΔ | *veA*1, *pabaA*1, *riboA*1, *nirA*637, pyroA4::*gpdA*_p_*-nirA*-*gfp::pyro3/4*, *msrA*Δ*::pyrG, msrB*Δ*::pyroA* (derived from a cross) | this work |
| *alcA*_p_-*FnirA* *msrAB*ΔΔ | *veA*1, *pabaA*1, *riboA*1, *nirA*637, *alcA*_p_*Flag*-*nirA*, *msrA*Δ*::pyrG, msrB*Δ*::pyroA* | this work |
| *kapK*^+^*–*S-Tag | *veA*1, *wa*3*, pyro*4*, argB*2*, pyrG*89*, nkuA*Δ*::argB, kapK*^+^*–*S-Tag*::pyrG* | (Bernreiter et al., 2007) |
| *alcA*_p_-*FnirA kapK^+^-*S-Tag | *veA*1, *wa*3*, pabaA*1*,* *pyroA*4*, nirA*637*::alcA*_p_*Flag*-*nirA, kapK*^+^*–*S-Tag::*pyrG* | this work |
| *alcA*_p_-*FnirA*^M169A^ *kapK^+^–*S-Tag | *veA*1, *wa*3*, pabaA*1*,* *pyroA*4*, nirA*637::*alcA*_p_*Flag*-*nirA*(M169A)*, kapK^+^–*S-Tag | this work |
| *hhoA*-*m-rfp* | *wA*2, *pabaA*1, *hhoA*-*m*-*rfp* (derived from LO1516) | (Nayak et al., 2010) |
| *gpdA_p_-nirA* ^NiRDΔ^-*gfp* | *veA*1, *pyroA*4, *riboB2*, *pyrG*89, *nirA*Δ::*riboB*fum, *gpdA*_p_*-nirA* ^NiRDΔ^*-gfp::pyr*4 (lacking amino acids 230 - 737, putative nitrate-regulatory domain NiRD) | this work |
