# Supplementary material for: Reversible Oxidation of a Conserved Methionine in the Nuclear Export Sequence Determines Subcellular Distribution and Activity of the Fungal Nitrate Regulator NirA
Source: PLoS Genet. 2015 Jul 1;11(7):e1005297. doi: 10.1371/journal.pgen.1005297 (PMC4488483; doi:10.1371/journal.pgen.1005297)
Supplement: S2 Table — Table shows changes in nirA nucleotide sequences in strains isolated as suppressors of the cold-sensitive nirA - strains. Numbers in the row “position” refer to amino acid positions within the altered proteins, mutations describe the amino acid changes caused by the nucleotide change given in the same lane and number of strains describes how many independent mutants were isolated carrying the described nucleotide change. (DOCX) [file pgen.1005297.s008.docx]

**Table S2. Nucleotide changes in the mutant strains.**

| **Position** | **Mutation** | **Nucleotide change** | **Nº strains** |
| --- | --- | --- | --- |
| 347 (CS) | Arg-Ser | CGC-AGC |  |
| 344 | Met-Ile | ATG-ATT  ATG-ATC  ATG-ATA | 1  1  3 |
| 342 | Ser-Arg | AGC-CGC | 4 |
| 315 | Lys-Glu | AAG-GAG | 3 |
| 312+310 | Val-Phe+Glu-Ala | GTC-TTC + GAG-GCG | 1 |
| 308 | Asn-Ile | AAC-ATC | 3 |
| 306 | Leu-Phe | CTT-TTT | 2 |
| 305 | Ile-Leu | ATC-CTC | 1 |
| 302 | Lys-Glu | AAG-GAG | 1 |
| 265 | Leu-Trp | TTG-TGG | 1 (TS) |
