# Supplementary material for: Reversible Oxidation of a Conserved Methionine in the Nuclear Export Sequence Determines Subcellular Distribution and Activity of the Fungal Nitrate Regulator NirA
Source: PLoS Genet. 2015 Jul 1;11(7):e1005297. doi: 10.1371/journal.pgen.1005297 (PMC4488483; doi:10.1371/journal.pgen.1005297)
Supplement: S3 Table — Table shows names and sequences of oligonucleotides used for cloning and biochemical experiments throughout this study. (DOCX) [file pgen.1005297.s009.docx]

**Table S3. Oligonucloetides used in this study**

| **Oligonucleotide** | **Sequence (5´to 3´)** |
| --- | --- |
| FMOA_UP_F | CAGAACTTGCATGTCGTCAG |
| FMOA_UP_R | CCTTCCTGGGCATCGCCTCT |
| FMOA_DOWN_F | ACATGAACATAGATGAAGACCG |
| FMOA_DOWN_R | CCAAGACCCCAGTACACTTC |
| FMOA_UP_NEST_F | CGGCGTATTTGTTGGGAGGT |
| FMOA_DOWN_NEST_R | GATGGAGATGCATAAGGGAC |
| FMOA_Pyro_F | TCTACCTCAATCATCTACGCTGAGAGGCGATGCCCAGGAAGGCTGCAGAAGTGCGCGAAAGCGTAAGGAGACCGGAAGC |
| FMOA_Pyro_R | GTTTTGACGGTCTTCATCTATGTTCATGTGGATCCAGGAGTATACGGGTTTTTGGCAT |
| FMOA_ORF_F | CATATTCAGTTTGAAACCCAGG |
| FMOA_ORF_R | GCAGGACTGGGAAAACAAGGGT |
| FmoB_Up_F | TTACCTGTCGTTGTTGGCCTATTAGAG |
| FmoB_Up_R | GCTGTCTGCTCTTGCTCTCCGCAGCAATGG |
| FmoB_Down_F | TTCAGGCAATAATACTCCGCAGCTAGATTATTATCAG |
| FmoB_Down_R | GCAGCCATGCTTGAAACACATACGCCATC |
| FmoB_Nest_Up_F | TCGACTACTGCTCAAGCTTTAATCGCTCCAG |
| FmoB_Nest_DOWN_R | ATAGATATGCCATGAGGTGAGGATCTTC |
| FMOB_Pyro_F | TCGGCTCGCTGACCATTGCTGCGGAGAGCAAGAGCAGACAGCCTGCAGAAGTGCGCGAAAGCGTAAGGAGACCGGAAGC |
| FMOB_Pyro_R | CACCGTATATAATATCTGATAATAATCTAGCTGCGGAGTATTATTGCCTGAAGGATCCAGGAGTATACGGGTTTTTGGCAT |
| FMOB_ORF_F | CGGAAGCAAAAGCGCGCTGGCCAG |
| FMOB_ORF_R | CATCCGTCACGAGAATTGTCTCGTCAGCC |
| nirAM169I_F | TGGAAAGATAAGCAACCTCGTGCTAGACGGATCCCGCAA |
| nirAM169I_R | TTGCGGGATCCGTCTAGCACGAGGTTGCTTATCTTTCCA |
| nirAC1_F | TCAATTCGAGTCTGAATTGGCTGTAAAGATGAGCAACCTC |
| nirAC1_R | GAGGTTGCTCATCTTTACAGCCAATTCAGACTCGAATTGA |
| nirAM169A_F | CTGAATTGGCTGGAAAGGCGAGCAACCTCGTGCTAGACG |
| nirAM169A_R | CGTCTAGCACGAGGTTGCTCGCCTTTCCAGCCAATTCAG |
| nirAC608A_F | agatgggagagtcttggcttgctgctcgccgga |
| nirAC608A_R | tccggcgagcagcaagccaagactctcccatct |
| pyroF | AAAGGCGAACATGTCGCTAGGAAG |
| URA-hER-pyro3/4_R | GGCCGCGAAGTTCACGACA |
| FMOB_*BspH* I_F | GAGAGAGATCATGACGCGCCCGCAAATTCG |
| FMOB_*Nco* I_R | TCTCTCTCCCATGGATGCACTGCACGCCGGTAG |
| niaD_F | GCAGCGGGCGGCTGCCAACTG-3 |
| niaD_R | TGATATAAAGTTAAAGATTCACGGCC |
| acnA_F | GATCGGTATGGGTCAGAAGGA |
| acnA_R | CGATGTTGCCGTACAGATCC |
| 18S_F | GAATGGCTCATTAAATCAGTTATCG |
| 18S_R | CGGGTTTAACCAGCTTTCCGGC |
| FMOB_Bgl II_Fwd | GAGAGAAGATCTGACGCGCCCGCAAATTCGACGAGTTG |
| FMOB_Not I_Rev | TCTCTCGCGGCCGCTGCACTGCACGCCGGTAGCACCGGA |
| NirA binding site 2* (NirA BS2*) | Biotin – TGAGCCGTGGCCAGACTTCCCAAATATCATCATTCGATCTCCAGCCCAACTCCGCGGAAATTCAGGCAGTGCATCGAAGCCATCCACGATGATCCACATCCACC |
| NirABS2 | GCCTGAATTTCCGCGGAGTTGGGCTGGA |
| BNirABS2i | Biotin-TCCAGCCCAACTCCGCGGAAATTCAGGC |
| nirAtruncAD_*Nco*I_Rev | TCTCTCTCCCATGGATGTTAGGTTCGGAGACGTACC |
| gpdA_F | TCGAGTACCATTTAATTCTATT |
| BaitI_fw | GATTTACCATGGGGGAAAG |
| BaitI_fus_rv | GTGCCTCAGTTGGGCTGTACGGACATAAGAGCC |
| BaitX_fus_fw | GGCTCTTATGTCCGTACAGCCCAACTGAGGCA |
| BaitX_rv | CTGCCATGGTGAATAGGCCTGCTATC |
