# Supplementary material for: Reversible Oxidation of a Conserved Methionine in the Nuclear Export Sequence Determines Subcellular Distribution and Activity of the Fungal Nitrate Regulator NirA
Source: PLoS Genet. 2015 Jul 1;11(7):e1005297. doi: 10.1371/journal.pgen.1005297 (PMC4488483; doi:10.1371/journal.pgen.1005297)
Supplement: S1 Text — (DOCX) [file pgen.1005297.s010.docx]

**Supporting Experimental Procedures and References**

**Construction of plasmids for gene expression in *A. nidulans*.** The plasmid *gpdA*_p_*-nirA*-GFP-*pyroA*3/4 was constructed by replacing the *pyr4* gene of the *gpdA*_p_*-nirA*-GFP plasmid [17] by a truncated *pyroA* gene amplified with the oligonucleotides “pyroF” and “URA-hER-pyro3/4_R”. The plasmid *gpdA*_p_*-fmoB*-GFP-*pyroA*3/4 was constructed by replacing the 2883 bp *NcoI* fragment containing the *nirA* ORF by a 1479 bp fragment containing the cDNA of the *fmoB* gene amplified after reverse transcription with oligonucleotides FMOB_*BspH* I_F and FMOB_*Nco* I_R. *gpdA*_p_*-nirA*^ADΔ^-GFP-*pyroA*3/4 was constructed by replacing the entire genomic *nirA* coding region (1-2676 bp) by a genomic *NcoI* fragment of 1-2097 bp of the *nirA* gene amplified with the oligonucleotides “gpdA_F” and “nirAtruncAD_*Nco*I_Rev” containing *NcoI* sites. The fragment codes for NirA amino acids 1-614 of NirA followed by an artificial serine derived from the cloning site and the start methionine of GFP. The plasmid gpdA_p_-*nirA*^NiRDΔ^-GFP-*pyr4* was constructed by the insertion in the *NcoI* restriction site of a fragment (1527 bp) obtained by the fusion of two PCR products called *nirA^I^* (expressing the NirA binding domain and NES, corresponding to amino-acids 1-229) and *nirA^X^* (expressing the NirA activation domain corresponding to amino-acids 738-892) [11] amplified by primers “BaitI_fw/BaitI_fus_rv” and by primers “BaitX_fus_fw/BaitX_rv” respectively (Table S2), using genomic DNA as template. The plasmid *alcA*_p_*-nirA* [17] carrying a FLAG-NirA fusion driven by the *A. nidulans alcA* promoter (designated in this work *alcA*_p_-*FnirA*) was used as a template for site directed mutagenesis of *nirA* generating the plasmids *alcA*_p_*-FnirA*^M169A^*, alcA*_p_*-FnirA*^M169I^ and *alcA*_p_*-FnirA*^c^1*.* The plasmid *gpdA*_p_*-nirA*-GFP-*pyroA*3/4 was used as a template for site directed mutagenesis generating *gpdA*_p_*-nirA*^M169A^-GFP-*pyroA*3/4. The plasmid ERE_p_-*nirA*-GFP [11] was used as a template for site directed mutagenesis to create ERE_p_-*nirA*^M169I^-GFP. All primers used for mutagenesis and PCR amplification are described in Supplementary Table ST 2. The site directed mutagenesis were performed with QuikChange® II Site-Directed Mutagenesis Kit (Stratagene) following the instructions of the provider. All constructs were sequenced in the final plasmids to avoid superfluous mutations.

**Construction of *fmoA* and *fmoB* deletion cassettes and generation of deletion strains.** In order to delete the ORF of the *fmoA* and the *fmoB* genes, deletion cassettes were constructed by DJ-PCR [60]. The deletion cassettes were constructed by replacing the ORF of *fmoA* and *fmoB* by *pyroA* flanked by the corresponding upstream and downstream sequences. 5´*fmoA* flanking sequence was amplified with oligonucleotides FMOA_UP_F and FMOA_UP_R, 3´*fmoA* flanking sequence was amplified with FMOA_DOWN_F and FMOA_DOWN_R. The *pyroA* gene containing overlapping sequences to 3´ and 5´ flanking sequences was amplified with FMOA_Pyro_F and FmoA_Pyro_R oligonucleotides. Nested primers FMOA_UP_NEST_F and FMOA_DOWN_NEST_R were used to amplify the complete assembled molecule.

5´*fmoB* flanking sequence was amplified with oligonucleotides FMOB_UP_F and FMOB_UP_R, 3´*fmoB* flanking sequence was amplified with FMOB_DOWN_F and FMOB_DOWN_R. The *pyroA* gene containing overlapping sequences to 3´ and 5´ *fmoB* ORF flanking sequences was amplified with FMOB_Pyro_F and FmoB_Pyro_R oligonucleotides. Nested primers FMOB_UP_NEST_F and FMOB_DOWN_NEST_R were used to amplify the complete assembled molecule. Wild type genomic DNA was used as a template in order to amplify all the fragments described above. The deletion cassettes were transformed into *kapK*1 [11] strain using standard procedures and the transformants were tested by PCR and Southern blot for the correct deletion events. The *alcA*_p_*-nirA fmoB*Δ strain was obtained after several crossing steps. The strain expressing *hhoA*-mRFP used for n-octylamine specificity testing was provided by Berl Oakley [61]

***E. coli* expression and purification of recombinant FmoB and activity measurements.** From wild type total RNA cDNA was generated by reverse transcription and by Phusion Flash High-Fidelity PCR (Finnzymes) using oligonucleotides FMOB_Bgl II_Fwd and FMOB_Not I_Rev (sequence details see Supplementary Table ST2) *fmoB* cDNA was amplified. The 1.5 kb fragment, lacking the stop codon, was inserted into BglII/NotI-digested pET-29a plasmid (Novagen) resulting in pET29a_*fmoB*, having a N-terminal (His)6-tag and C-terminal S-tag. FmoB protein was expressed in *E. coli* BL21 (DE3) pLysS cells using Overnight Express Instant TB Medium (Novagen). Cells were resuspended in 15 ml sonication buffer (100 mM sodium phosphate buffer pH 8.0, 150 mM NaCl) amended with bacterial protease inhibitor cocktail (Sigma) and sonicated. Cell debris was removed by centrifugation and the supernatant passed through a 0.45 µm filter. All following chromatography steps were performed at 4°C. Extracts were loaded on 2.5 ml His-Select Nickel Affinity Gel resin (Sigma) washed with sonification buffer containing 5 mM imidazole and eluted by 30 and 60 mM imidazole. FmoB containing fractions were purified to homogeneity by size exclusion chromatography on a Superdex 200 PG 16/60 column (GE Healthcare) using 50 mM phosphate buffer pH 8.0, 50 mM NaCl as eluent, and aliquots stored at -80°C.

The photometric NADPH assay contained 112 µg purified FmoB protein, aerated 50 mM phosphate buffer pH 8.0, 50 mM NaCl, 0.2 mM NADPH, and different substrates at various concentrations (methimazole, L - methionine, L - methionine sulfoxide) in 1 ml total volume. The components were mixed and the reaction was started by adding FmoB protein. NADPH oxidase activity of FmoB in the absence or presence of substrate [62] (methimazole) was monitored over time by following the decrease in absorption (340 nm). For HPLC analysis the reaction volume was 0.5 ml, containing 56 µg FmoB protein, aerated 50 mM phosphate buffer pH 8.0, 50 mM NaCl, ^+^/_-_ 0.4 mM NADPH, ^+^/_-_ 5 mM L – methionine. As before, the reaction was started by the addition of FmoB protein and the mixture was incubated at 25°C for up to 300 seconds. Subsequently L - norvalin was added as internal standard before derivatization with o-phthalaldehyde and analysis by RP-HPLC according to a published procedure [63]

**Supporting material references**

60. Yu, J.H., Hamari, Z., Han, K.H., Seo, J.A., Reyes-Dominguez, Y., and Scazzocchio, C. (2004). Double-joint PCR: a PCR-based molecular tool for gene manipulations in filamentous fungi. Fungal genetics and biology : FG & B *41*, 973-981.

61. Nayak, T., Edgerton-Morgan, H., Horio, T., Xiong, Y., De Souza, C.P., Osmani, S.A., and Oakley, B.R. (2010). Gamma-tubulin regulates the anaphase-promoting complex/cyclosome during interphase. J Cell Biol *190*, 317-330.

62. Alfieri, A., Malito, E., Orru, R., Fraaije, M.W., and Mattevi, A. (2008). Revealing the moonlighting role of NADP in the structure of a flavin-containing monooxygenase. Proc Natl Acad Sci U S A *105*, 6572-6577.

63. Altmann, F. (1992). Determination of amino sugars and amino acids in glycoconjugates using precolumn derivatization with o-phthalaldehyde. Anal Biochem *204*, 215-219.
